# Cross-talk of gut-bone-muscle: osteosarcopenia in experimental colitis models

**DOI:** 10.1186/s42826-026-00273-x

**Published:** 2026-04-14

**Authors:** Shilpa Sharma

**Affiliations:** Department of Medicine, The University of Melbourne, Melbourne, Australia

**Keywords:** Inflammatory bowel disease, Colitis, Osteosarcopenia, Musculoskeletal deficits, Mechanistic targets, Gut-derived serotonin, Muscle damage, Therapeutics

## Abstract

Inflammatory bowel disease (IBD) represents a group of chronic inflammatory conditions that extend beyond the gastrointestinal tract, with profound systemic effects on the musculoskeletal system. This review comprehensively examines the experimental models that have elucidated the intricate relationship between intestinal inflammation and the development of osteosarcopenia—the concurrent deterioration of bone and muscle tissues. We analyze chemically-induced models (DSS, TNBS), genetically engineered models (T-cell transfer, IL-10 knockout, IL-2 knockout), and the Winnie mouse model of spontaneous colitis, highlighting their contributions to our understanding of IBD-associated musculoskeletal complications. The review emphasizes how these models have revealed critical molecular mediators, particularly gut-derived serotonin and vitamin D signaling pathways, that connect intestinal pathology to distant skeletal deterioration. Notably, pro-inflammatory cytokines including TNF-α, IL-6, and IL-17 emerge as central regulators affecting both bone and muscle homeostasis, explaining the synchronized pattern of deterioration observed clinically. The findings from these experimental models highlight potential therapeutic targets and intervention strategies beyond conventional approaches, including gut-derived serotonin inhibition and vitamin D modulation. This review underscores the value of chronic colitis models in elucidating the complex pathophysiology of IBD-associated osteosarcopenia and provides direction for translational research to develop integrated treatment approaches for this debilitating extraintestinal manifestation.

## Background

Inflammatory bowel disease (IBD), comprising Crohn’s disease (CD) and ulcerative colitis (UC), represents a group of chronic and relapsing inflammatory disorders primarily affecting the gastrointestinal tract. Global prevalence has risen to approximately 0.5–0.8% of the population, with the highest rates observed in North America and Europe, though incidence is increasing worldwide, particularly in newly industrialized countries [1, 2]. This rise reflects IBD’s emergence as a global disease associated with significant morbidity, impaired quality of life, and substantial healthcare costs.

Although the exact etiology remains elusive, IBD is understood to develop through complex interactions between genetic susceptibility, environmental triggers, immune system dysregulation, and alterations in gut microbiota [3]. The pathophysiology involves excessive mucosal immune responses against luminal antigens in genetically predisposed individuals, leading to chronic inflammation, tissue damage, and architectural distortion of the intestinal wall. This inflammatory cascade is characterized by overproduction of pro-inflammatory cytokines, including tumor necrosis factor-alpha (TNF-α), interleukin-1β (IL-1β), IL-6, and IL-17, which orchestrate and perpetuate the inflammatory response.

Growing evidence establishes IBD not merely as a localized intestinal disorder but as a systemic inflammatory condition with numerous extraintestinal manifestations (EIMs) affecting multiple organ systems. EIMs occur in up to 40–50% of IBD patients, with higher prevalence in CD than UC, and can develop before, during, or after the diagnosis of intestinal disease [4]. Among these, musculoskeletal complications represent the most common category of EIMs, affecting approximately 20–40% of IBD patients [5]. Osteoporosis affects approximately 13–42% of IBD patients, resulting in a fracture risk 40% higher than the general population [6, 7]. Similarly, sarcopenia occurs in 40–60% of IBD patients [8]. This integrated bone-muscle relationship is particularly prevalent in IBD, where patients often experience concurrent bone loss (osteopenia/osteoporosis) and muscle wasting (sarcopenia), a condition recently termed “osteosarcopenia” [9].

**Fig. 1:**
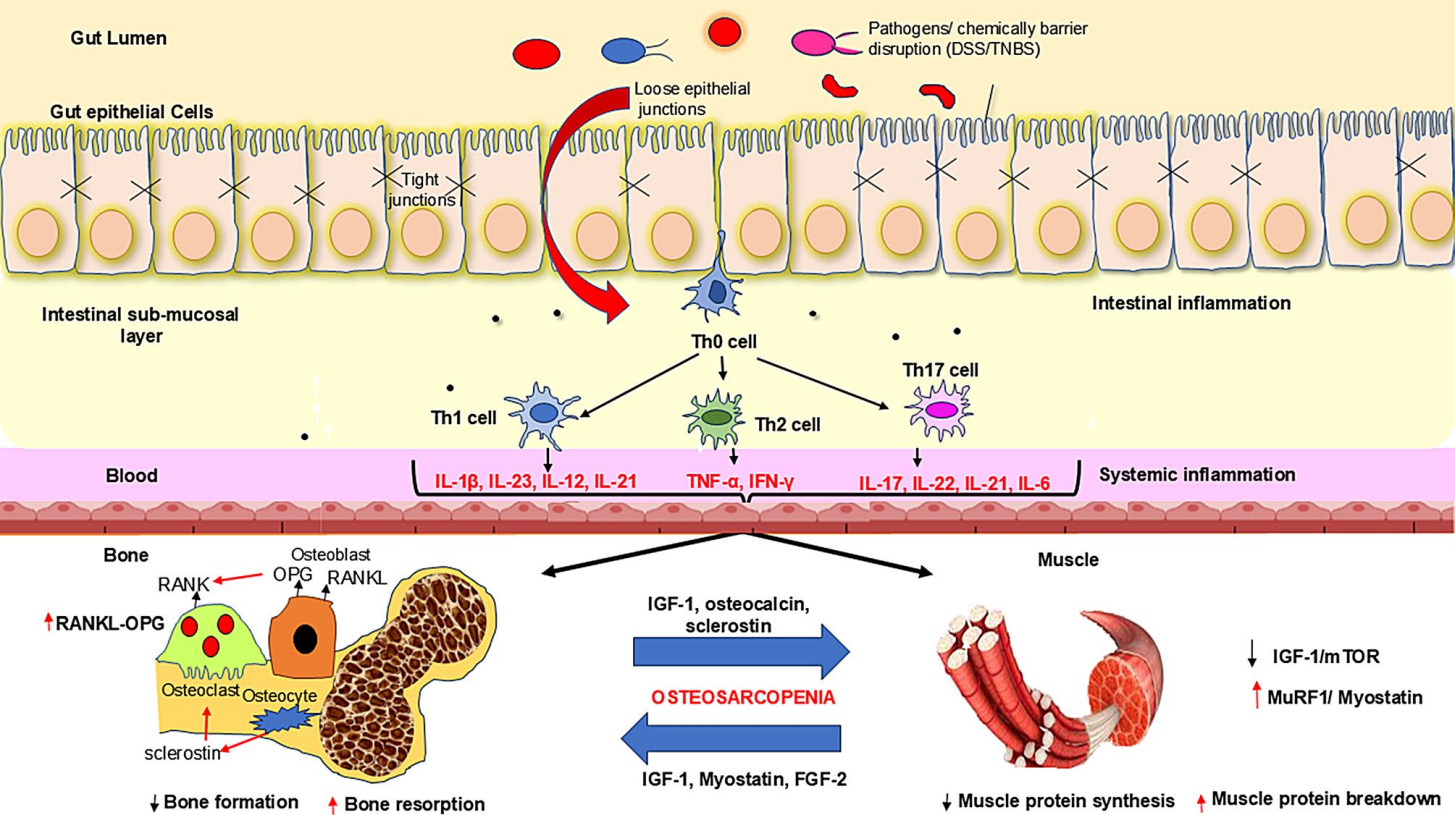
Pathways connecting gut inflammation to skeletal health. Figure illustrates the pathogenesis of osteosarcopenia in IBD. Intestinal barrier disruption allows luminal antigens to penetrate the lamina propria, activating immune cells. Naive T helper cells differentiate into pro-inflammatory subsets that secrete various inflammatory cytokines. These mediators enter systemic circulation, affecting distant tissues, particularly bone and muscle. In bone tissue, circulating inflammatory cytokines disrupt the balance between formation and resorption. This occurs primarily through modulation of the RANK/RANKL/OPG pathway, with downregulation of osteoblast markers and upregulation of osteoclastogenic factors. Higher RANKL to OPG ratio drive bone resorption in the course of IBD. Concurrently in muscle tissue, the inflammatory environment disrupts protein homeostasis, favoring catabolism over synthesis. A reciprocal relationship exists between bone and muscle deterioration, where factors released from one tissue negatively impact the other, creating a cycle of progressive osteosarcopenia

While systemic inflammation contributes to both bone and muscle deterioration in IBD, compelling evidence supports the existence of a specific gut-bone-muscle axis with directional, mechanistic pathways connecting these tissues beyond generalized inflammatory effects. The journey from intestinal inflammation to osteosarcopenia in IBD follows a fascinating multi-stage cascade that traverses multiple organ systems through shared inflammatory pathways. This process begins within the intestinal ecosystem, where dysbiosis creates the initial inflammatory trigger, disrupting tight junction proteins (e.g., occludin, claudin, zonula occludens-1), compromising the epithelial barrier [10]. Upon breaching the epithelial barrier, microbial antigens encounter antigen-presenting cells, which process and present them to naïve T helper (Th0) cells, initiating a cascade that ultimately impacts both bone and muscle through specific molecular mechanisms rather than merely systemic inflammation (Fig. 1).

The gut microbiome emerges as a central orchestrator in this gut-bone-muscle axis, providing a foundation for tissue-specific cross-talk. IBD patients exhibit hallmark disruptions in microbial ecology—decreased microbial diversity, increased Gram-negative bacteria, and diminished Gram-positive populations [11]. This dysbiotic signature, characterized by depleted beneficial *Faecalibacterium prausnitzii* and enriched potentially pathogenic *Enterobacteriaceae*, creates a fundamental shift from protective to pro-inflammatory microbial functions [12, 13]. Beyond triggering intestinal inflammation, these microbial changes directly impact skeletal tissues through microbial metabolites, bacterial translocation, and altered immune signaling, establishing the microbiome as a pivotal mediator in the gut-bone-muscle axis rather than merely an intestinal factor.

The microbiome communicates with distant skeletal tissues through multiple pathways. In healthy conditions, microbiota-derived short-chain fatty acids (SCFAs), particularly butyrate, support intestinal barrier integrity, suppress inflammation, promote osteoblast growth while inhibiting osteoclastogenesis, foster immunoregulatory T cell development, and enhance muscle insulin sensitivity [14, 15]. SCFAs improve muscle function by upregulating insulin-responsive glucose transporter type 4 expression through adenosine monophosphate-activated protein kinase activation, enhancing energy utilization [16]. The dysbiosis associated with IBD disrupts this beneficial SCFA production, simultaneously affecting both bone and muscle metabolism through shared signaling pathways.

The compromised intestinal barrier allows bacterial translocation of microbiota-derived products—particularly lipopolysaccharide (LPS)—which activates toll-like receptors (TLR4) and NOD-like receptors in both local and distant tissues. This triggers NF-κB signaling pathways that upregulate production of pro-inflammatory cytokines IL-1β, IL-6, and TNF-α [17]. These inflammatory mediators enter systemic circulation, creating a state of chronic low-grade inflammation that simultaneously affects multiple organ systems [18]. The activation of TLR4 signaling in both osteoclasts and myocytes directly contributes to osteosarcopenia through enhanced bone resorption and muscle catabolism [19].

The receptor activator of nuclear factor kappa B (RANK), receptor activator of nuclear factor kappa B ligand (RANKL), and osteoprotegerin (OPG) signaling axis provides some of the most compelling evidence for an integrated gut-bone-muscle axis, as this system demonstrates direct mechanistic connections between bone and muscle tissues. While initially characterized as a primary regulator of bone metabolism, the RANK/RANKL/OPG axis has now been demonstrated to function directly in muscle tissue, where it regulates protein turnover and energy homeostasis [20]. Studies of OPG-deficient mice reveal a striking dual phenotype of both osteoporosis and muscle atrophy, confirming shared regulatory mechanisms across these tissues rather than parallel but independent effects of systemic inflammation [21].

The molecular evidence establishing RANK/RANKL/OPG signaling in muscle protein degradation is particularly robust. Skeletal muscle fibers express functional RANK receptors, and RANKL binding activates downstream cascades that ultimately influence protein turnover, energy homeostasis, and inflammatory responses [22]. RANKL signaling in muscle directly activates NF-κB, a master regulator of muscle catabolism, leading to upregulation of the ubiquitin-proteasome system—the primary pathway for protein degradation in skeletal muscle [23]. This activation specifically increases expression of muscle-specific E3 ubiquitin ligases MuRF1 and MAFBx/Atrogin-1, which target myofibrillar proteins for degradation [24], providing a direct mechanistic link between RANKL signaling and muscle protein breakdown.

In inflammatory conditions like IBD, elevated cytokines like TNF-α, IL-1β, and IL-6 stimulate RANKL expression while simultaneously suppressing OPG in both bone and muscle tissues. This shift in the RANKL/OPG ratio creates a systemic environment that promotes catabolism in both tissues through parallel mechanisms [20]. Crucially, muscle-specific deletion of RANK generates protection against denervation-induced muscle atrophy while improving specific force in mice [21], confirming that RANK signaling directly impacts muscle physiology independently of its effects on bone. Despite these significant advances in understanding the RANK/RANKL/OPG axis in bone-muscle cross-talk, this pathway remains virtually unexplored specifically in the context of IBD-associated muscle wasting.

The bone-muscle cross-talk extends beyond the RANK/RANKL/OPG axis to include bidirectional communication through numerous additional signaling mechanisms. The shared inflammatory burden in IBD disrupts the normal communication between bone and muscle through interference with the production and signaling of myokines and osteokines, further exacerbating the deterioration of both tissues [25]. This integrated communication network includes paracrine and endocrine molecular signaling mediators such as myostatin, transforming growth factor-β, bone morphogenetic proteins, activin, follistatin, insulin-like growth factor 1 (IGF-1), fibroblast growth factor, osteoglycan, osteonectin, irisin, monocyte chemoattractant protein, and matrix metallopeptidases [26], creating multiple avenues for coordinated regulation of both tissues.

This concept of an integrated “gut-bone-muscle axis” represents a paradigm shift in understanding IBD-associated musculoskeletal complications, moving beyond traditional organ-specific approaches or general systemic inflammation to recognize the coordinated nature of these tissues and their communication through shared signals and molecular mediators. The directional flow from intestinal dysbiosis and inflammation to altered microbiome-derived metabolites to specific effects on both bone and muscle through shared signaling pathways demonstrates the existence of this axis as a defined pathophysiological entity rather than merely parallel manifestations of systemic inflammation.

This review examines the pathophysiological mechanisms connecting intestinal inflammation with bone and muscle deterioration in IBD. We begin by discussing experimental colitis models that have provided valuable insights into these connections, followed by exploration of key mediators of the gut-bone-muscle axis: gut-derived serotonin, and vitamin D signaling. Finally, we explore emerging therapeutic approaches that target these pathways, including conventional pharmaceutical interventions, promising natural compounds, and novel targeted therapies with dual benefits for both intestinal and musculoskeletal manifestations. By elucidating these molecular pathways and their therapeutic implications, we aim to provide a comprehensive framework for understanding and treating IBD-associated osteosarcopenia.

## Main Text

### Experimental models of colitis

Experimental models of colitis provide valuable platforms for investigating the complex relationship between intestinal inflammation and musculoskeletal health [27, 28]. These models span a spectrum of complexity, from chemically-induced acute inflammation to genetically engineered models of spontaneous chronic disease. By examining this range, we can build a comprehensive understanding of how different aspects of IBD pathophysiology contribute to skeletal deterioration. Table 1 summarizes the key experimental colitis models discussed in this review, including the species, gender, and age of animals used; colitis induction protocols; principal skeletal and muscle findings. Colitis models have demonstrated anassociation between chronic intestinal inflammation and musculoskeletal deterioration, pointing to complex inflammatory and immune-mediated mechanisms beyond simple nutritional deficits [27, 29]. The following sections examine each colitis model in detail, focusing on their specific contributions to our understanding of the gut-bone-muscle axis in IBD.

**Table 1:** Musculoskeletal effects in experimental models of colitis

Model	Species/ Gender/Age	Colitis Protocol	Key Skeletal Findings	Key Muscle Changes
DSS [30]	BALB/C male, 9 weeks old	5% DSS in drinking water for 1 week + 2.5% DSS for another week	Trabecular bone loss, enhanced osteoclastogenesis, minimal cortical effects	Not assessed
DSS [31]	C57BL/6 male, 4 weeks old	5% DSS for 5 days, assessed at 7, 15, 29, and 43 days following 1st day of DSS administration.	Reduced trabecular BMD, bone volume, thickness; cortical thinning; reduced osteoblast markers	Not assessed
DSS [32]	C57BL/6 male, 5–6 weeks old	1% DSS for 15 days	Decreased femoral bone mass, reduced osteoblast markers, unaffected osteoclast activity	Not assessed
DSS [33]	Sprague Dawley male, 8 weeks old	2% DSS for 4 weeks	Decreased BMD, reduced cancellous bone volume, lower ultimate load capacity, increased osteoclast surface	Not assessed
DSS [34, 35]	Piglets, 5–7 days old	1–5% DSS, 50% macronutrient restricted diet compared with thoseharma receiving probiotics or adequate nutrition and healthy well-nourished controls	Reduced BMD in cortical/trabecular bone, decreased bone integrity regardless of nutrition	Preserved muscle protein synthesis with adequate nutrition; impaired mTOR signaling with restricted nutrition
DSS [36]	C57BL/6 N male, 10 weeks old	3% DSS for 5 days), 2% DSS for 5 days)	Not assessed	Reduced muscle weight, fiber size, protein content; downregulated IGF1-R and mTOR; overexpression of atrophy markers (Murf-1, Myostatin)
DSS [37]	C57BL/6 male, 8 weeks old	2.5% DSS for 8 days	Not assessed	Activation of Erk signaling in skeletal muscle; increased myh7 gene expression; fiber-type transitions from fast-to-slow twitch
TNBS [38]	Sprague Dawley rats, male, 8–9 weeks old	Rats were given repeated rectal administrations of TNBS (30 mg/kg in 30% ethanol) on days 1, 7, 14, 21, and 26.	Cancellous bone volume loss, increased osteocyte TNF-α and IL-6, elevated sclerostin	Not assessed
TNBS [39]	Male C57Bl/6 mice, 7 weeks old	TNBS/ ethanol enema	Not assessed	Muscle atrophy from increased protein degradation rather than decreased synthesis; upregulated MuRF-1 and atrogin-1; elevated TNF-α, IL-6, and NOS2 expression
T-cell transfer [40]	CB6F1, mice aged 12–14 weeks to female C.B.17 scid/scid mice 14–16 weeks old	Transfer of CD4^+^CD45RB^Hi^ T cells isolated form CB6F1 mice (donors) to scid mice recipients	Decreased bone density, inflammatory bone marrow infiltrate, altered PTH levels	Not assessed
IL-10 KO [41]	Female IL-10^−/−^ mice, 9 weeks old	Piroxicam for 12 days	Reduced trabecular thickness, elevated serum CTX, increased colonic RANKL and OPG	Not assessed
IL-10 KO [42]	C57BL/6, 14 week old, male and female IL-10^−/−^ mice	C57BL/6 infected with *H. hepaticus* and examined 6 weeks later	Reduced femur/vertebrae trabecular bone volume, greater bone loss in males	Not assessed
IL-2 KO [43]	C57BL/6, 6 week old	CD4 T cells from C57BL/6 IL2^−/−^ or IL2^+/+^ (donor) injected to 6 week old C57BL/6-*Rag1*^*−/−*^, assessed at 6–8 weeks	Comprehensive bone loss affecting both cortical and trabecular compartments	Not assessed
*Winnie* [44, 45]	Male and female C57BL/6 mice, 6, 15, 24 weeks old	Missense mutation (G9492A) in the Muc2 gene, resulting in aberrant mucin production and natural development of intestinal inflammation (79,80)	Progressive bone deterioration with age; reduced osteoblast numbers and collagen synthesis at 6 weeks; severe microarchitecture compromise at 24 weeks	Reduced muscle mass, decreased fiber size, impaired functional endurance; changes correlate with inflammatory markers

### Chemically-induced models of colitis

#### Dextran sodium sulfate-induced colitis model

The dextran sodium sulfate (DSS) colitis model stands as the most commonly employed experimental system for investigating UC pathogenesis and potential interventions [46]. DSS, chemical colitogen with anticoagulant properties, which acts directly on the colonic epithelium, disrupting barrier function and triggering an innate immune response [47]. Mechanistically, DSS forms complexes with medium-chain fatty acids in the colonic lumen, creating nanovesicles that integrate with colonocyte membranes and induce damage primarily in the distal colon [46]. The DSS-induced colitis model has gained widespread adoption due to its reproducibility, ease of implementation, and recapitulation of key human IBD features. However, an important limitation is that while luminal bacteria contribute significantly to colitis development, this model does not require adaptive immunity to induce inflammation [48]. DSS-induced inflammation occurs independently of T and B cells, as it can be induced in severe combined immunodeficiency (SCID) mice, making it primarily an innate immunity-driven model. The DSS model exhibits a biphasic response. The acute phase (days 1–7) is characterized by epithelial damage, neutrophil infiltration, and production of pro-inflammatory cytokines, including IL-1β, TNF-α, and IL-6. Clinical manifestations include weight loss, diarrhea, rectal bleeding, and shortened colon length [27]. In the recovery/chronic phase after DSS withdrawal, a repair process begins, featuring epithelial restitution, crypt regeneration, and a shift toward adaptive immune responses with increased T cell involvement. Repeated cycles of DSS administration can lead to chronic inflammation with fibrosis and dysplasia [27]. Histologically, DSS-induced colitis is characterized by erosion and ulceration of the epithelium, crypt loss and distortion, goblet cell depletion, massive mucosal and submucosal infiltration of inflammatory cells, edema and occasional crypt abscesses [48]. The inflammation typically begins in the distal colon and progresses proximally, similar to the distribution pattern observed in human UC [48, 49].

Low molecular weight DSS induces more severe inflammation in the cecum and upper colon, while higher molecular weight DSS affects primarily the distal colon. DSS concentration typically ranges from 1 to 5% (w/v) in drinking water, with higher concentrations causing more severe disease [50]. Acute models typically employ 5–7 days of DSS administration, while chronic models use multiple cycles of DSS with recovery periods [48].

DSS stimulates acute inflammation by activating Th1/Th17 pathways, resulting in increased production of TNF-α, IL-6, IL-17, and keratinocyte-derived chemokines. Chronic colitis is characterized by persistent inflammation, prominent adaptive immune involvement (T and B cell responses), fibrosis, and incomplete recovery between cycles leading to progressive disease [50]. The chronic state switches to Th2-mediated inflammation, established by amplified production of anti-inflammatory IL-4 and IL-10 and decreased production of the inflammatory cytokines [49].

Recent applications have expanded to examining gut microbiota’s role in IBD development and assessing how dietary factors and genetic background influence disease severity. A landmark comparative study using conventional, germ-free (GF), and antibiotic-treated “pseudo-germ-free” (PGF) mice revealed unexpected differences in DSS response across these groups [51]. Counterintuitively, GF mice exhibited minimal inflammation but more severe epithelial damage and bleeding, while PGF mice with subtotal microbiota depletion showed an attenuated yet relatively normal inflammatory response with enhanced barrier function, correlating with increased IL-10 and Foxp3 expression. These findings demonstrate the microbiota’s dual role in DSS colitis—both driving inflammatory responses and maintaining epithelial integrity—suggesting that microbiota-targeted interventions require careful consideration. Further complicating this relationship, recent evidence indicates that DSS exposure alone causes negligible alterations to microbial taxonomy and functionality without host involvement, emphasizing that DSS-induced pathology primarily reflects host-microbiota interactions rather than direct microbial perturbation [52].

The genetic background of mouse strains significantly influences DSS susceptibility, providing valuable insights into human IBD heterogeneity. BALB/c mice, with their Th2-biased immune profile, demonstrate remarkable resistance to DSS challenge characterized by reduced pro-inflammatory cytokines (IFN-γ and TNF-α) balanced by elevated anti-inflammatory mediators and expanded regulatory T cell populations [53]. In contrast, C57BL/6 mice exhibit heightened vulnerability due to their predominant Th1-polarized immunity. Importantly, even within genetically identical strains, colitis susceptibility can vary substantially based on vendor source, with microbiota composition emerging as a determinant potentially as significant as genetic background. This concept is supported by observations that BALB/c mice harboring higher abundances of Actinobacteriota and butyrate-producing Roseburia species demonstrate enhanced resistance to colitis development [54].

#### Bone effects in DSS-induced colitis

The skeletal consequences of DSS-induced colitis are substantial, with meta-analysis confirming significant deterioration of both trabecular and cortical bone architecture, decreased bone formation rates, and strong correlations between inflammatory cytokine levels and bone loss [55]. These findings are supported by robust correlations between intestinal inflammation severity and bone structural parameters across multiple DSS-induced colitis studies. Hamdani et al. investigated the impact of DSS-induced acute colitis on skeletal health in male BALB/c mice [30]. DSS colitis mice displayed significant osteopenia characterized by comprehensive trabecular bone deterioration, including decreased femoral trabecular bone volume, trabecular number, trabecular surface, mineralizing surface, bone formation rate, and growth plate thickness. Notably, while trabecular compartments showed marked changes, cortical bone parameters remained relatively unaffected [30]. The study further demonstrated enhanced osteoclastogenesis in DSS-treated mice, suggesting increased bone resorption as a key mechanism driving bone loss. Similarly, excessive osteoclast formation was reported in DSS-induced colitis in Sprague Dawley male rats [56]. Another study revealed that colitis induces an expanded population of osteoclast precursor cells in bone marrow, with enhanced osteoclastogenic potential and upregulate myeloid DNAX activation protein 12-associating lectin-1 (MDL-1), a pro-osteoclastogenic co-receptor [57]. These findings highlight a specific cellular mechanism linking intestinal inflammation to skeletal deterioration through pathologic expansion and functional reprogramming of osteoclast progenitors.

In a complementary investigation, study evaluated the temporal effects of acute colitis on developing skeletal tissue by administering DSS to male C57BL/6 mice [31]. During active colitis, substantial reductions in trabecular bone mineral density (BMD), bone volume, and thickness were observed. Cortical bone parameters were similarly affected, with decreased thickness, outer perimeter, and density, alongside increased inner perimeter and marrow area. These skeletal changes correlated strongly with intestinal inflammation severity and significant body weight reduction, the latter likely resulting from decreased nutritional intake during active disease. The temporal relationship between inflammatory cytokine profiles and skeletal parameters is particularly revealing—as TNF-α and other inflammatory mediators rise during active colitis, osteoblastogenic markers (RUNX2, alkaline phosphatase, osteocalcin) become suppressed during the acute phase, only to normalize when inflammation resolves, highlighting the direct influence of gut-derived inflammatory signaling on distant skeletal tissues. Remarkably, by days 29 and 43, these molecular markers normalized in parallel with recovery of bone parameters and body weight, highlighting the potential reversibility of inflammation-induced bone pathology when intestinal inflammation resolves [31].

Irwin et al. investigated bone alterations caused by moderate intestinal inflammation by administering a low dose of DSS to male C57BL/6 mice for 15 days [32]. The DSS-induced colitis mice exhibited severe body weight and bone loss. While these mice lost substantial subcutaneous fat, they accumulated more visceral fat. The bone loss manifested as distorted bone microarchitecture and significantly decreased femoral bone mass. The decline in osteoblast markers (osteocalcin and RUNX2) suggested that reduced osteoblast proliferation was the primary cause of bone loss. The researchers also observed decreased growth plate thickness and reduced collagen X, a hypertrophic chondrocyte matrix component. Increased TNF-α levels in both colon and bone tissue correlated with decreased bone mass. Notably, osteoclast activity remained unaffected by DSS-induced colitis. Importantly, the DSS model demonstrates that inflammation-induced bone loss can be reversible, as both intestinal healing and skeletal recovery occur following DSS withdrawal. This reversibility parallels clinical observations in IBD patients, where effective control of disease activity can lead to partial recovery of BMD [32].

Metzger et al. (2019) investigated how DSS (2%) induced chronic colitis affected bone cellular and mechanical properties to male Sprague Dawley rats [33]. While their DSS colitis model showed elevated gut histopathology scores, the inflammatory response was more moderate compared to the TNBS colitis model. The chronic DSS-induced colitis in young rats led to several significant changes, decreased body weight, reduced BMD, lower cancellous bone volume, and reduced ultimate load capacity. These skeletal changes were accompanied by increased osteoclast surface area, decreased bone formation rate, and elevated levels of pro-inflammatory cytokines in osteocytes, including TNF-α, IL-6, RANKL, and OPG [33].

Vassilyadi et al. (2016) investigated if preserving appropriate nutritional status could safeguard bone integrity despite ongoing colitis in developing piglets (5–7 day old) [34]. The researchers divided the piglets into two dietary groups. One group received 100% of macro- and micronutrient requirements. The other group received 50% of macronutrients but 100% of micronutrient requirements. Findings in piglets with colitis included reduced areal and volumetric BMD in cortical and trabecular bone, decreased cortical area, trabecular area, and bone mineral content, resulting in bone fragility. Notably, these bone alterations occurred regardless of nutritional status, leading researchers to conclude that colitis impaired bone structure and strength independently of nutrient intake [34].

This integrated analysis of the DSS model illustrates how intestinal inflammation creates a systemic inflammatory environment that affects skeletal tissues through both shared inflammatory mediators and tissue-specific mechanisms. The differential responses of bone and muscle to nutritional intervention during active inflammation have important therapeutic implications, suggesting bone preservation in IBD may require direct anti-inflammatory interventions, while muscle maintenance might be achievable through optimized nutritional support even during ongoing intestinal inflammation. These findings highlight the value of comprehensive approaches addressing both primary intestinal pathology and its systemic consequences for optimal management of IBD-associated osteosarcopenia.

#### Muscle effects in DSS-induced colitis

Inflammatory pathways simultaneously impact skeletal muscle, though with intriguing tissue-specific differences. Studies in DSS-treated male mice (background C57BL/6 N) reveal significant sarcopenia development characterized by reduced muscle weight, fiber size, and protein content [36]. Analysis of molecular pathways revealed comprehensive alterations characteristic of IBD-related sarcopenia, including downregulation of muscle growth markers (IGF1-R and mTOR) and overexpression of atrophy markers (Murf-1 and Myostatin). The muscles exhibited lower global protein content, likely resulting from increased protein degradation via the E3 ligases MuRF1/Atrogin1/MAFbx axis and FOXO3 [36]. Additionally, inflammatory signals like IL-23 activate Erk signaling in skeletal muscle, driving fiber-type transitions from fast-to-slow twitch, represent another mechanism through which inflammatory signals directly reprograms muscle phenotype in DSS treated male C57BL/6 mice [37].

The relationship between inflammation and nutrition reveals a fascinating tissue-specific dichotomy in DSS model. Studies in piglets demonstrate that while colitis directly impairs bone integrity regardless of nutritional status, muscle protein metabolism remains largely protected when adequate nutrition is maintained [34, 35]. Colitis with adequate nutrition preserves muscle protein synthesis rates and mTOR signaling despite stunted growth, while macronutrient restriction during colitis dramatically impairs translation initiation. This suggests bone metabolism is primarily driven by inflammation-activated pathways operating independently of nutrition, while muscle maintains anabolic capacity during inflammation provided sufficient nutritional substrates are available.

#### Trinitrobenzene sulfonic acid-induced colitis model

Mice exposed to trinitrobenzene sulfonic acid (TNBS) develop widespread transmural inflammation throughout the colon (pancolitis), which more closely mirrors the pathology seen in CD. TNBS acts as a hapten, binding to colonic proteins and rendering them immunogenic [58]. This creates a predominantly Th1 and Th17-driven inflammatory response, closely mimicking the cytokine profile observed in human CD [28]. Single rectal instillation of TNBS dissolved in ethanol causes acute colitis, which peaks at 3–7 days post-administration. This is predominantly innate immune infiltration initially, followed by T-cell responses. Repeated administration of TNBS at lower doses with intervals between treatments causes chronic colitis. Oh et al. (2014) compared inflammatory mechanisms in 4% DSS-induced (acute), 2% DSS-induced (chronic), and 2% TNBS-induced acute models of colitis [28]. All 3 mouse models of colitis showed an increase in the number of IFN-γ^+^ and IL-17^+^ T cells and a decrease in the number of IL-4^+^ T cells [28]. Mice with 2% DSS-induced colitis (chronic) indicated recurrent oscillations in weight loss during the 30-day experimental period, similar to the progression of IBD in humans [28].

#### Bone effects in TNBS-induced colitis

TNBS-treated male Sprague Dawley rats provide compelling evidence that chronic intestinal inflammation profoundly affects bone biology through osteocyte-mediated mechanisms. Following just four weeks of experimental colitis, TNBS colitis model exhibited significant cancellous bone volume loss concurrent with extensive intestinal epithelial damage. The inflammatory cascade within bone tissue revealed striking alterations in osteocyte function. Osteocytes in TNBS-induced colitis mice showed approximately higher expression of TNF-α and higher IL-6 levels, establishing a pro-inflammatory microenvironment in distant skeletal sites. This inflammatory state directly impacted bone remodeling through increased RANKL and OPG expression in osteocytes. Most notably, the study revealed that osteocyte sclerostin, a potent inhibitor of bone formation, increased significantly in response to inflammation, with nearly 60% of this increase predicted by osteocyte TNF-α levels. These elevations in sclerostin strongly correlated with decreased bone formation parameters, illuminating a direct mechanistic pathway connecting gut inflammation to impaired bone formation through osteocyte-mediated signaling [38].

Beyond direct effects on bone cells, TNBS colitis disrupts mineral homeostasis through inflammatory cytokine actions on calcium regulatory mechanisms. Elevated TNF-α and IFN-γ in colitis mice downregulate the expression and activity of Klotho, an anti-inflammatory protein crucial for renal calcium homeostasis [59]. This mechanism helps explain how intestinal inflammation in IBD can rapidly impact skeletal health through disruption of calcium homeostasis, independent of nutritional factors or prolonged disease duration.

While osteocyte dysfunction and calcium dysregulation primarily affect BMD, intestinal inflammation also impairs skeletal growth through endocrine disruption. Studies in pre-pubertal male TNBS-induced colitis mice showed that approximately one-third of growth impairment stems directly from inflammation through a separate endocrine pathway. The mechanism involves inflammation-induced hepatic growth hormone resistance, creating a paradoxical relationship between GH and IGF-1 [60]. While nutritional deficits elevate GH as expected, inflammation disrupts this compensation, significantly reducing circulating IGF-1. Exogenous IGF-1 partially rescues growth in colitic but not pair-fed animals, indicating inflammation targets hepatic IGF-1 production rather than causing growth plate resistance [60]. This endocrine disruption represents a third mechanism through which intestinal inflammation compromises skeletal integrity, particularly during critical developmental periods.

The complex interplay between these mechanisms—osteocyte-mediated inflammation, calcium homeostasis disruption, and growth hormone resistance—collectively orchestrates bone deterioration in IBD. These findings significantly advance our understanding of IBD-associated bone loss by demonstrating that multiple physiological systems are simultaneously compromised. Osteocytes are not merely passive responders to circulating inflammatory signals but active participants in propagating and amplifying inflammatory cascades within the bone microenvironment. This multifaceted pathophysiology suggests that optimal bone health in IBD requires comprehensive approaches combining nutritional support with targeted anti-inflammatory interventions addressing each of these distinct but interconnected pathways.

When interpreting these findings, it’s important to consider methodological context. Though chemical agents like TNBS are widely used and provide valuable insights, they create non-specific and erosive intestinal defects that may not perfectly recapitulate human IBD [61]. Their typically self-limiting course may underestimate the cumulative bone effects of chronic, long-term inflammation seen in human IBD patients [62]. Emerging genetically engineered models that develop spontaneous, chronic intestinal inflammation may provide additional insights into the prolonged effects of inflammation on bone integrity, particularly regarding mechanisms that might amplify or mitigate bone loss over extended periods.

#### Muscle effects in TNBS-induced colitis

The TNBS colitis model elegantly demonstrates how intestinal inflammation triggers muscle atrophy through specific molecular mechanisms. At its core, increased protein degradation—rather than decreased synthesis—drives muscle loss through enhanced proteasome activation and upregulation of muscle-specific atrogenes MuRF-1 and atrogin-1. This predominance of catabolic processes over anabolic dysfunction represents a critical insight for therapeutic targeting in IBD-associated sarcopenia. The catabolic environment develops within a complex inflammatory milieu characterized by elevated TNF-α, IL-6, and NOS2 expression in both liver and skeletal muscle tissues, accompanied by increased circulating levels of these inflammatory cytokines [39, 58].

The preservation of protein synthesis rates despite significant proteolysis provides important mechanistic insights with therapeutic implications. First, it distinguishes IBD-associated muscle wasting from simple malnutrition, where both protein synthesis and degradation are typically affected. Second, it suggests that targeted therapies inhibiting protein degradation pathways might be particularly effective for addressing IBD-associated sarcopenia, while nutritional interventions alone may have limited efficacy. Third, it highlights how inflammatory signals originating in the intestine can directly reprogram muscle metabolism at distant sites through specific molecular pathways rather than through generalized systemic effects [39].

### Genetically engineered models: revealing immune-mediated mechanisms

#### T cell transfer model: insights into adaptive immunity

Building on insights from chemical models, researchers developed more sophisticated approaches to simulate the immune dysregulation characteristic of human IBD. Specifically, when donor-derived naive T cells expand in immunodeficient recipients, they induce transmural colitis with severe inflammation throughout the small intestine [63]. Recipient strains must be genetically identical to the donor strain to prevent graft-versus-host disease.

The intestinal microbiome plays a critical role in determining disease outcomes in this model. The presence of commensal gut microbiota, particularly segmented filamentous bacteria (SFB), significantly impacts recipient susceptibility to various immune-related outcomes, including the success of T-cell transfer. SFB influences the maturation and differentiation of T cells, which can profoundly affect therapeutic outcomes [64]. A key advantage of this adoptive T cell transfer model is that it enables researchers to investigate the immunological basis of intestinal inflammation from initial onset through advanced disease stages.

In IBD, hyperactivated CD4^+^ T lymphocytes secrete abnormally high levels of proinflammatory cytokines, driving intestinal inflammation. T cell adoptive transfer models have proven invaluable not only for elucidating the critical role of T cells in maintaining intestinal homeostasis, but also for characterizing the specific contributions of various T cell subpopulations to colitis pathogenesis.

#### Bone effects in T cell transfer model

An elegant adoptive transfer model reveals how specific T cell populations directly trigger bone deterioration in IBD [40]. Mice receiving CD4^+^CD45RB^Hi^ T cells developed simultaneous colitis and osteopenia with decreased bone density and disrupted bone cell balance, despite maintaining normal food intake. The bone marrow microenvironment in these animals showed infiltration of TNF-α-producing inflammatory cells and altered parathyroid hormone levels, creating a hostile environment for bone formation [40]. Remarkably, osteoprotegerin treatment effectively reversed established bone loss and prevented its development without affecting gut inflammation, providing proof that skeletal deterioration can occur through inflammatory pathways independent of malabsorption. This selective bone protection through RANKL inhibition suggests a promising therapeutic strategy for IBD patients, addressing skeletal fragility without necessarily modifying intestinal disease.

A specialized CD4^+^ T cell subset—Th17 cells co-expressing TNF-α accumulate in the bone marrow during active intestinal inflammation, creating a destructive environment through dual mechanisms: direct expression of RANKL and indirect stimulation of bone marrow stromal cells (BMSCs) to produce osteoclastogenic factors. IL-17 produced by Th17 cells upregulates RANKL expression on stromal cells and induces MCP-1 (monocyte chemoattractant protein-1) and MIP-1α (macrophage inflammatory protein-1α), thereby recruiting osteoclast precursors [65]. TNF-α further impairs bone formation by downregulating osteoblast development through upregulation of E3 ubiquitin ligase (Smurf1), which reduces critical bone formation transcription factors, Runx2 and Smad1 [65]. Maintained by IL-7 from BMSCs, these pathogenic T cells establish an inflammatory niche that perpetuates bone loss [66]. Recently, it has been reported that IL-17 cytokine family serves dual roles in immunity—providing essential protection against pathogens while potentially driving destructive inflammation [67].

#### IL-10 knockout colitis model

Expanding on the role of specific immune components, cytokine knockout models further refined our understanding of the molecular mediators involved in IBD pathogenesis. The IL-10 knockout (IL-10^−/−^) model demonstrates that deficiencies in anti-inflammatory signaling pathways can lead to spontaneous intestinal inflammation without external chemical triggers. IL-10 deficiency creates an inflammatory cascade by simultaneously promoting pathogenic Th1/Th17 expansion and impairing protective Treg function [68].

This immunological imbalance drives a distinct inflammatory signature characterized by elevated pro-inflammatory cytokines (TNF-α, IL-17, IL-6) and enhanced NF-κB signaling that extends beyond intestinal pathology to directly impact musculoskeletal function [69]. While both the T cell transfer and IL-10^−/−^ models recapitulate chronic inflammation, the IL-10^−/−^ model offers the particular advantage of spontaneous disease development without requiring cell transfer procedures, providing insights into how deficiencies in endogenous anti-inflammatory pathways contribute to IBD pathogenesis.

The pathophysiology of IL-10^−/−^ mice can be further manipulated through microbial and pharmacological interventions. Enterohepatic Helicobacter species infection, particularly *H. hepaticus*, triggers enhanced colitis development in these immunocompromised mice, demonstrating microbiota’s causal role in mediating systemic effects of intestinal inflammation. Remarkably, *H. hepaticus* becomes the dominant microbiota member within 14 days post-infection, inducing robust Th1 and Th17 immune responses marked by elevated IFN-γ and IL-17 levels [70]. This combined model (*H. hepaticus* infected IL-10^−/−^ mice) provides valuable insights into gender-specific aspects of IBD-associated musculoskeletal complications and the role of specific cytokine pathways.

In IL-10^−/−^ mice, the onset and severity of spontaneous colitis varies considerably depending on mouse strain, background, and host microbial composition [71]. Yang and colleagues demonstrated this variability when identical C57BL/6J IL-10^−/−^ mice housed under specific pathogen-free conditions at two different facilities showed dramatically different responses to *Helicobacter hepaticus* infection - one group developed robust typhlocolitis while the other showed minimal inflammation, suggesting that microbiota differences were the critical determinant of disease susceptibility [72].

Piroxicam administration accelerates and standardizes the colitis phenotype in IL-10^−/−^ mice by disrupting prostaglandin-mediated epithelial protection, resulting in severe CD-like pathology with intestinal hyperplasia and focal transmural inflammation. This pharmacologically-enhanced model creates a reliable experimental system for investigating therapeutic interventions targeting inflammation-induced bone loss [41].

#### Bone effects in IL-10 ^−/−^ models

IL-10^−/−^ mice with colitis exhibit profound bone deterioration characterized primarily by suppressed bone formation rather than increased resorption. This is evidenced by decreased mineralizing surfaces in cancellous bone, reduced serum osteocalcin, and impaired mineralized nodule formation in bone marrow cultures [73]. The direct relationship between inflammation and skeletal deterioration is confirmed by the observation that IL-10^−/−^ mice with colitis show significantly greater bone loss than those without colitis, with bone mechanical properties becoming compromised through reduced stiffness and lower fracture thresholds.

The underlying molecular mechanisms involve a cascade of inflammatory mediators. Pro-inflammatory cytokines like TNF-α directly inhibit Cbfa1/Runx2, the master regulator of osteoblast differentiation, while both TNF-α and IFN-γ trigger increased nitric oxide production through iNOS activation. This elevated NO creates a hostile environment for osteoblasts, inhibiting their function and promoting their apoptosis. Reduced serum IGF-1 further compromises bone formation, particularly in animals with severe colitis [73].

The *H. hepaticus*-infected IL-10^−/−^ model further illuminates these pathways, demonstrating significant trabecular bone loss in both femur and vertebrae alongside modest cortical thinning in both sexes [42]. Analysis of bone markers in male mice reveals suppressed osteoblast activity, consistent with previous findings showing reduced bone formation in various IBD mouse models. This suppression of bone-forming cells appears to be a primary driver of bone loss rather than increased bone resorption. The severity of cecal inflammation (disease score) negatively correlates with bone volume in male mice, indicating that the inflammatory process directly impacts bone metabolism. Systemic inflammation involving Th1 and Th17 immune responses, with male mice showing increased interferon gamma (IFN-γ) and IL-17 levels in the gut. Inflammatory cytokines that likely travel from the inflamed intestine to bone tissue, impairing osteoblast function [42].

Perhaps most intriguing is the consistent sexual dimorphism observed across multiple studies - male IL-10^−/−^ mice consistently show more pronounced bone loss than females despite comparable intestinal inflammation [41, 74], highlighting the complex interplay between inflammatory signaling and sex-specific factors in skeletal regulation.

Recent transcriptomic analysis has revealed a critical role for disrupted Wnt signaling in this pathology. In male IL-10^−/−^ BALB/c mice induced with piroxicam, lower bone formation rates were observed along with reduced osteoblastogenesis and higher adipogenesis of bone marrow mesenchymal stromal cell [75]. Intestinal inflammation appears to reprogram these stromal cells by downregulating Wnt pathway genes, creating a direct molecular bridge between gut inflammation and skeletal stem cell fate decisions. TNF-α emerges as a central mediator of this effect, interfering with Wnt-responsive gene expression and redirecting stromal cells toward adipogenesis rather than osteoblastogenesis.

The piroxicam-accelerated colitis model in female IL-10^−/−^ mice (C57BL/6J background) has provided additional mechanistic insights, revealing that enhanced bone resorption through the RANKL-RANK-OPG axis complements impaired formation in driving bone loss. Serum CTX levels inversely correlate with trabecular thickness, while the inflamed colon shows significant upregulation of both RANKL and OPG alongside increased pro-inflammatory cytokines and T-cell activation markers [41]. This dual disruption of bone homeostasis - simultaneously impairing formation while enhancing resorption - creates a “perfect storm” for skeletal deterioration that closely parallels human IBD pathophysiology [76].

#### IL-2 knockout model

Mice deficient in IL-2 develop chronic colonic inflammation at 8–9 weeks of age that strikingly resembles human UC. In this model, disease onset depends on colonization with commensal microbes, highlighting the essential interplay between genetic susceptibility and environmental triggers [77].

Sadlack’s original description showed attenuated colitis in specific pathogen-free conditions and initially no evidence of disease in GF IL-2-deficient mice, suggesting luminal bacteria provide the persistent antigenic stimulus for colitis development [78]. However, longer-term studies by Schultz revealed that older GF IL-2-deficient mice (up to 46 weeks) eventually develop mild, focal intestinal inflammation, possibly triggered by bacterial constituents in autoclaved food [79].

Interestingly, subsequent research demonstrated that colonization with even a single bacterial species could induce colonic inflammation in these mice. While microbial exposure primarily affects intestinal pathology, other manifestations including anemia, lymphoid hyperplasia, and hepatic inflammation occur independently of bacterial colonization [80].

Beyond its value in studying intestinal pathology, this model has provided groundbreaking insights into the molecular mechanisms connecting gut inflammation and bone deterioration.

#### Bone effects in IL-2^−/−^ model

The IL-2^−/−^ model provides a valuable perspective on inflammation-induced bone loss. Skeletal deterioration begins just at 4 weeks—before significant intestinal inflammation is apparent—and progressively worsens at 7 weeks (moderate) and 9 weeks (severe colitis) [43]. This temporal sequence reveals that subtle immunological dysregulation impacts skeletal homeostasis even before overt intestinal pathology develops, suggesting that bone may serve as an early indicator of underlying immune disturbances.

Comprehensive analysis shows that bone loss affects both cortical and trabecular compartments simultaneously, creating a pattern of skeletal deterioration that closely mirrors findings in human IBD patients. Elegant adoptive transfer experiments have definitively established T cells as the central mediators of this bone loss. When CD3^+^ T cells from IL-2^−/−^ mice are transferred to lymphocyte-deficient C57BL/6-Rag1^−/−^ recipients, they faithfully reproduce both intestinal inflammation and skeletal deterioration, resulting in decreased femoral BMD, reduced trabecular volume, suppressed osteoblastogenesis, and enhanced osteoclastogenesis [43].

The molecular mechanism underlying this gut-bone axis centers on RANKL production by autoreactive T cells. This multifunctional molecule serves as the critical bridge between intestinal and skeletal pathology—simultaneously promoting bone resorption through osteoclast activation and perpetuating intestinal inflammation. The broader inflammatory milieu—characterized by elevated TNF-α, IL-1β, and IL-6—creates a perfect storm for skeletal deterioration by simultaneously activating osteoclasts, inhibiting osteoblasts, and disrupting normal bone remodeling [43]. When combined with malabsorption of calcium and vitamin D due to intestinal damage, these mechanisms collectively produce a skeletal phenotype that provides an ideal platform for studying targeted therapeutic approaches to IBD-associated osteoporosis and testing interventions that might protect bone without necessarily modifying intestinal disease.

#### Winnie mice model of colitis: bridging epithelial dysfunction and chronic inflammation

In the landscape of experimental colitis models, the development of *Winnie* mice represents a fascinating departure from traditional approaches. Rather than relying on chemical induction or targeted gene knockouts, these mice emerged through N-ethyl-N-nitrosourea mutagenesis—a method that introduces random, heritable point mutations through chemical means [81, 82]. This approach allows researchers to discover novel phenotypes without preconceived genetic targets.

The *Winnie* mouse, carrying a missense mutation in the MUC2 mucin gene, serves as a valuable model for UC, exhibiting mucus barrier dysfunction and spontaneous intestinal inflammation. These mice demonstrate key features of human IBD, including mucosal damage, goblet cell depletion, increased intestinal permeability, and altered colonic innervation [81, 83–85]. These mice exhibit a complex inflammatory profile involving both innate and adaptive immune responses, with a striking 100-fold increase in IL-17 A production that establishes a dominant IL-23/Th17 response. This immunological landscape, featuring elevated levels of activated dendritic cells, and enhanced secretion of pro-inflammatory cytokines, creates a remarkably authentic recapitulation of human IBD pathology—all stemming from a single, random mutation that altered mucin production and compromised intestinal barrier function [86].

Wang et al. investigated whether microbiota is essential for colitis development in this model by comparing germ-free and conventionally housed *Winnie* mice [87]. Their findings revealed a two-step pathogenic process: mucin misfolding in intestinal goblet cells initiates mild inflammatory signaling even without microbiota (as evidenced by persistent chemokine elevation), while the microbiota serves as a critical amplifier establishing progressive inflammation. RNA sequencing demonstrated that *Winnie* colon organoids have decreased expression of negative inflammatory regulators, making epithelial cells more responsive to inflammatory stimuli. This research conclusively shows that epithelial protein misfolding can prime inflammation independently, but microbiota is necessary for full colitis amplification. Tala et al. revealed that the intestinal environment, genetically determined by the MUC2 mutation, selects for a microbial community with specific pro-inflammatory, genotoxic, and metabolic properties that contribute to chronic inflammation [88]. Together, these studies illuminate the complex interplay between genetic susceptibility, protein misfolding, and microbiota in IBD pathogenesis, showing how epithelial dysfunction creates both intrinsic inflammatory signals and a permissive environment for microbiota-driven inflammation amplification.

#### Bone effects in the Winnie model: temporal progression of skeletal deterioration

The *Winnie* mouse model reveals a fascinating timeline of skeletal deterioration that begins subtly to profound architectural compromise. Studies in *Winnie* mice have revealed severity-dependent bone deterioration that correlates with intestinal inflammation, providing a valuable window into the relationship between gut pathology and skeletal health [44].

Even at the transitional 6-week stage, when intestinal inflammation appears clinically mild, the skeletal system already shows the first whispers of pathology—declining osteoblast numbers, reduced collagen synthesis, and suppressed bone formation signal the earliest casualties of the inflammatory assault [44].

By 14 weeks, as intestinal disease intensifies, these initial deficits evolve into a comprehensive skeletal crisis. The bone’s microarchitecture undergoes dramatic remodeling with thinning of both cortical and trabecular compartments. The cellular imbalance becomes pronounced with significantly reduced osteoblastogenesis occurring alongside a paradoxical surge in osteoclast numbers at the metaphysis—the metabolically active region where bone turnover is most dynamic. Ex vivo bone marrow analysis showed that *Winnie* mice had low mineralized nodules, characteristic of low matrix formation [44].

Bone deterioration reaches its zenith at 24 weeks in *Winnie* mice, where the most severe skeletal compromise is observed. This accelerated decline likely reflects the cumulative damage from sustained inflammatory insult and the progressive failure of compensatory mechanisms. This temporal intensification mirrors the clinical reality in human IBD, where bone loss correlates with both disease duration and cumulative inflammatory burden. At this advanced stage, the bone’s fundamental building blocks are dramatically diminished, with significant reductions in osteoid volume, collagen I expression, and total collagen content creating a structurally compromised skeleton that bears the full signature of chronic IBD [44]. This temporal progression illustrates how IBD creates a hostile skeletal environment, with cellular and matrix abnormalities preceding measurable architectural changes. The *Winnie* model thus captures the insidious nature of inflammatory bone loss—a process that begins with subtle cellular imbalances before manifesting as the clinically recognized complication of IBD-associated osteopenia and osteoporosis.

#### Muscle effects in the Winnie model

The *Winnie* mouse model demonstrates progressive muscle alterations that parallel intestinal inflammation, characterized by reduced muscle mass, decreased fibre size, and impaired functional endurance. Muscle changes correlate significantly with disease activity and inflammatory markers, establishing a direct relationship between intestinal inflammation and muscle wasting at inflammatory stage at 15 weeks [45]. This study establishes the *Winnie* mouse as a valuable model for investigating IBD-related muscle wasting, providing opportunities to develop targeted interventions for preventing and treating muscle complications in IBD patients.

#### Muscle-bone interaction in experimental colitis

Colitis models reveal that beyond sharing common inflammatory drivers, these tissues engage in cross-talk through specialized signaling molecules. This communication network extends beyond biochemical signaling to include mechanical influences, as inflammation-induced alterations affect both tissues, though often with distinct temporal patterns and responses to interventions.

- Experimental models across the spectrum—from acute chemical induction (DSS, TNBS) to genetic models (IL-10^−/−^, *Winnie*)—demonstrate that both tissues respond to systemic inflammatory signals, with bone showing particular sensitivity to cytokine-mediated disruption of remodeling processes. The activation of shared catabolic pathways through NF-κB signaling, coupled with suppression of anabolic IGF-1/mTOR signaling in both tissues, creates a unified metabolic profile that explains the clinical co-occurrence of sarcopenia and osteopenia in IBD patients.
- The integrated analysis of the DSS model illustrates how intestinal inflammation creates a systemic inflammatory environment that affects skeletal tissues through both shared inflammatory mediators and tissue-specific mechanisms. The differential responses of bone and muscle to nutritional intervention during active inflammation have important therapeutic implications, suggesting bone preservation in IBD may require direct anti-inflammatory interventions, while muscle maintenance might be achievable through optimized nutritional support even during ongoing intestinal inflammation.
- These findings highlight the value of comprehensive approaches addressing both primary intestinal pathology and its systemic consequences for optimal management of IBD-associated osteosarcopenia. This integrated understanding suggests that therapeutic strategies targeting either tissue alone may yield suboptimal outcomes, while approaches addressing the muscle-bone unit holistically could provide synergistic benefits in preserving musculoskeletal integrity during chronic intestinal inflammation.

#### Age-related considerations in experimental colitis models

A significant limitation of the current literature is that most experimental colitis studies utilize relatively young mice (4–10 weeks of age), corresponding to adolescence or early adulthood in humans. Only the *Winni*e mouse model has been studied at more advanced ages, with bone effects characterized at 24 weeks, revealing dramatic reductions in osteoid volume, collagen expression, and fundamental bone architecture [44]. However, even this represents only middle age in murine lifespan, with truly aged models (> 18 months) remaining largely unexplored in the context of IBD-associated musculoskeletal complications.

The susceptibility to experimentally induced colitis shows significant age-dependent variation. Recent studies demonstrated that DSS administration induced more severe colitis, with aged animals exhibiting higher disease activity indices, more extensive tissue damage, and prolonged recovery periods [89]. This age-dependent susceptibility appears to be mediated through decreased intestinal barrier integrity, with aged mice showing reduced expression of tight junction proteins (occludin, claudin-1) at baseline and more pronounced disruption following inflammatory challenge [89]. The basal inflammatory state of the intestine changes with age, with older animals exhibiting higher constitutive expression of pro-inflammatory cytokines and greater numbers of activated resident immune cells, creating a primed environment that responds more vigorously to colitis-inducing agents [89]. Additionally, microbiota composition changes significantly with aging, with older animals showing reduced diversity and beneficial commensal species that normally provide colonization resistance against pathobionts [89]. These age-dependent differences in colitis induction directly impact the subsequent development of musculoskeletal complications.

Young animals possess robust compensatory mechanisms including greater bone formation capacity, enhanced muscle regenerative potential, and more resilient nutrient absorption even during active inflammation, which may partially offset the catabolic effects of intestinal inflammation. In contrast, aged animals likely experience accelerated and more severe musculoskeletal deterioration due to reduced baseline bone mass, impaired osteoblast function, enhanced inflammatory sensitivity, and accumulated senescent cell populations that amplify inflammatory responses.

Recent multi-omic investigations reveal that age-dependent cytokine expression patterns likely contribute to the increased susceptibility of elderly IBD patients to osteosarcopenia [90]. While younger individuals maintain higher levels of protective mediators like LTA, older subjects show elevated expression of vascular endothelial growth factor A (VEGFA)—a cytokine identified as a shared risk factor for both bone and muscle deterioration. The complex tissue-specific effects of certain inflammatory signals—beneficial for one tissue while detrimental to another—suggest that age-related changes in this delicate balance could disrupt musculoskeletal homeostasis in older IBD patients.

Future research should extend existing colitis models into aged mice to better reflect elderly IBD patients while conducting comparative studies of young versus aged animals using identical colitis induction protocols. Such studies should assess whether anti-inflammatory or anti-senescence interventions show differential efficacy across age groups. Understanding these age-specific mechanisms would provide critical insights for developing personalized therapeutic strategies targeting the unique needs of IBD patients across different life stages, particularly for elderly populations facing both age-related and inflammation-induced musculoskeletal deterioration simultaneously.

### Gut-derived serotonin: a critical molecular mediator in the gut-bone axis

Examining specific molecular mediators helps elucidate the mechanisms underlying the gut-bone axis in IBD. Among these mediators, gut-derived serotonin (GDS) has emerged as a pivotal molecular messenger translating intestinal inflammation into skeletal pathology [91]. This sophisticated signaling pathway reveals how systemic consequences of intestinal inflammation extend far beyond the gut.

**Fig. 2:**
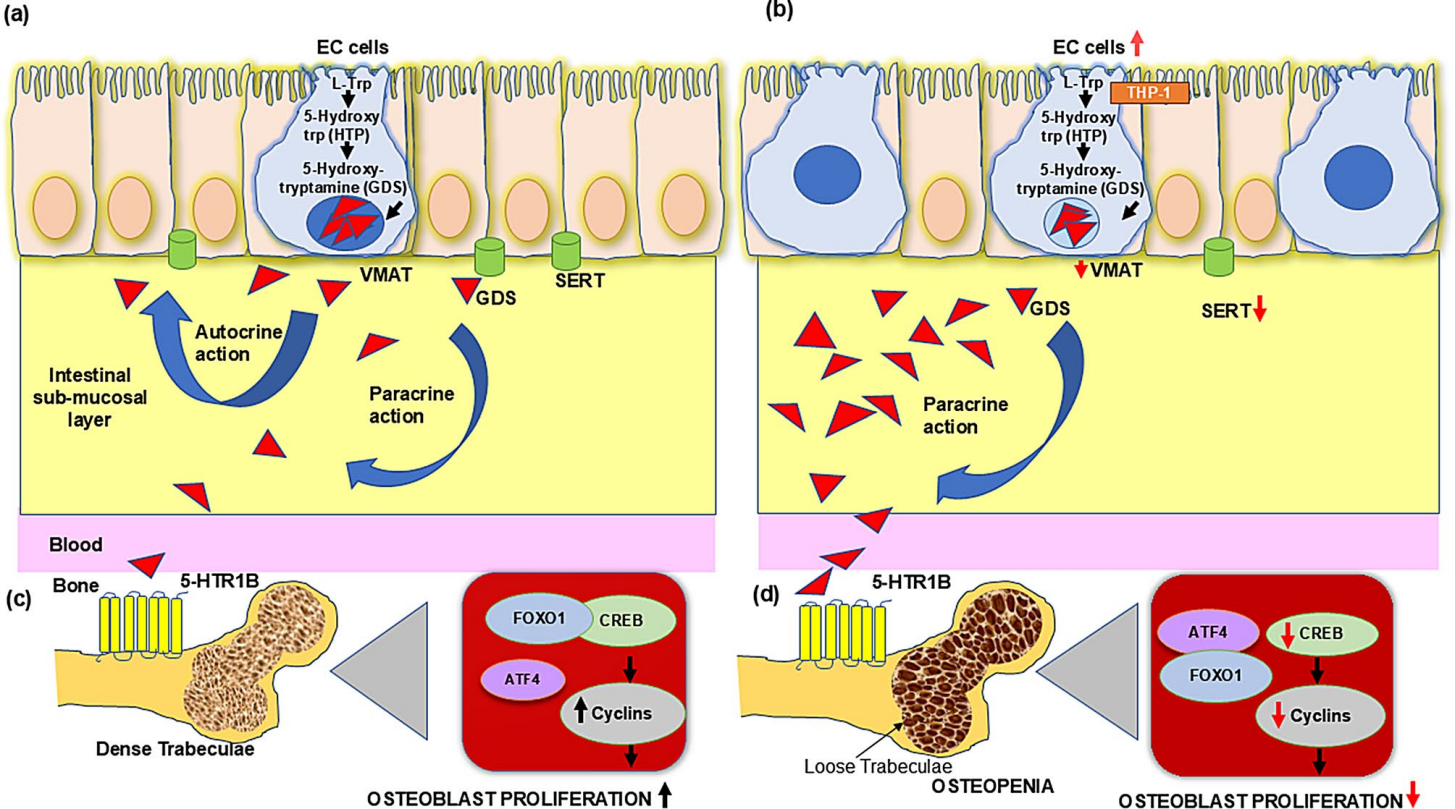
Differential regulation of gut-derived serotonin (GDS) signaling and its impact on bone metabolism in healthy versus osteopenic conditions. (**a**) In healthy conditions, enterochromaffin cells in the intestinal epithelium synthesize GDS from L-tryptophan (L-Trp) through the rate-limiting enzyme tryptophan hydroxylase (THP-1). GDS is stored in vesicles and released at normal levels. The serotonin reuptake transporter (SERT) efficiently regulates GDS concentrations in the intestinal submucosal layer through reuptake mechanisms. Moderate GDS levels reach bone tissue through both autocrine and paracrine signaling pathways, where they interact with 5-HTR1B receptors on osteoblasts. This balanced signaling maintains healthy bone characterized by dense trabecular structure. (**b**) In osteopenic conditions (such as during chronic intestinal inflammation), there is an increased number of enterochromaffin cells, reduced SERT expression, leading to impaired GDS reuptake and elevated circulating GDS. The increased GDS levels excessively activate 5-HTR1B receptors on osteoblasts, resulting in a pathological bone phenotype characterized by loose trabecular structure consistent with osteopenia. (**c**) The molecular mechanism in healthy bone involves FOXO1 and CREB transcription factors forming a functional complex that promotes cyclin expression, leading to normal osteoblast proliferation. (**d**) In osteopenic conditions, the molecular signaling is disrupted. FOXO1 dissociates from CREB and associates with ATF4, resulting in suppressed cyclin expression and, consequently decreased osteoblast proliferation. This model demonstrates how altered gut-derived serotonin signaling represents a key molecular mechanism linking intestinal inflammation to skeletal abnormalities in conditions such as inflammatory bowel disease

In physiological conditions (Fig. 2a), enterochromaffin cells within the intestinal epithelium synthesize serotonin through a well-regulated pathway. L-tryptophan is converted to 5-hydroxytryptophan by the rate-limiting enzyme tryptophan hydroxylase-1 (THP-1), and subsequently to serotonin (GDS) [92]. This process is tightly controlled, with GDS being packaged into vesicles via the vesicular monoamine transporter (VMAT) and released at precisely regulated levels. Critically, the serotonin reuptake transporter (SERT) expressed in intestinal epithelial cells maintains homeostatic GDS concentrations through efficient reuptake mechanisms [93]. This balance ensures that only moderate levels of GDS reach the circulation, where it exerts both autocrine and paracrine effects on target tissues.

GDS effects on bone metabolism involve specific molecular mechanisms. GDS inhibits osteoblast proliferation by binding to 5-HTR1B receptors on pre-osteoblasts [94, 95], initiating a signaling cascade that ultimately suppresses osteoblast proliferation and bone formation. This inhibitory effect is supported by compelling evidence showing that bone formation increases when TPH1 is inhibited [96], and that pharmacological intervention with 5-HTR1B antagonists promotes bone formation by blocking GDS’s inhibitory effects [97].

During chronic intestinal inflammation (Fig. 2b), this finely tuned system becomes dysregulated. The *Winnie* mouse model reveals a previously underappreciated connection between GDS and progressive bone deterioration in IBD. Both circulating serotonin levels and disease activity show a linear progression with age, establishing GDS as a more reliable indicator of ongoing disease evolution.

Inflammatory mediators alter enterochromaffin cell function while significantly downregulating SERT expression, severely compromising GDS reuptake as observed in *Winnie* mice from 7 to 24 weeks [98]. Consequently, elevated GDS levels flood the circulation, creating a pathological signaling environment. Studies have documented elevated GDS levels in both TNBS- and DSS-induced colitis models across multiple species [99, 100]. Experimental studies in mice have demonstrated that increased GDS levels inhibit autophagy pathways, thereby enhancing vulnerability to colitis development and progression [101]. The significance of GDS in colitis pathogenesis was further demonstrated using TPH1-deficient mice, which lack the rate-limiting enzyme for peripheral serotonin synthesis. These knockout mice show attenuated colitis severity, which can be reversed by administering 5-hydroxytryptophan to restore serotonin production [102], providing mechanistic evidence for GDS’s direct involvement in inflammatory processes.

This temporal correlation between GDS levels and bone microarchitecture illuminates a potential mechanistic pathway. In healthy bone, GDS signaling through 5-HTR1B receptors initiates a cascade where transcription factors FOXO1 and CREB form a functional complex that promotes appropriate cyclin expression, maintaining normal osteoblast proliferation (Fig. 2c). However, during pathological conditions, increased GDS oversaturates 5-HTR1B receptors, disrupting this signaling pathway. This manifests through a critical shift where excessive 5-HTR1B signaling causes FOXO1 to dissociate from CREB and instead form a complex with ATF4 (Fig. 2d). The interaction between these two molecular regulators, combined with increased binding of GDS to its bone-specific receptor (5-HTR1B), creates a perfect storm that suppresses osteoblast function and bone formation.

Lavoie et al. have provided direct experimental evidence for this mechanism, demonstrating the detrimental effect of elevated GDS on bone in the DSS mouse model of colitis. Their work revealed that colitis-induced bone loss was characterized by reduced trabecular bone volume, decreased osteoblast proliferation, and enhanced bone resorption [103]. Importantly, they showed that lowering GDS levels with its antagonist, parachlorophenylalanine, as well as inhibition of the 5-HTR1B receptors provided a protective effect against colitis-induced bone changes [103]. These findings establish a clear causal relationship between elevated GDS and skeletal pathology in experimental colitis.

The bidirectional relationship depicted in Fig. 2 has profound clinical implications. It demonstrates that bone loss in IBD represents more than just a consequence of malnutrition, vitamin D deficiency, or glucocorticoid therapy—it reflects a direct molecular pathway through which intestinal inflammation affects skeletal metabolism. Importantly, disease severity correlates with GDS levels, suggesting this pathway plays a dose-dependent role in mediating inflammation-associated bone loss.

This serotonergic signaling axis represents an elegant molecular bridge connecting intestinal inflammation to skeletal deterioration—one that operates independently of direct inflammatory cytokine effects. The discovery that serotonin levels track closely with bone deterioration highlights the importance of looking beyond the gut wall to understand the systemic manifestations of IBD. This molecular dialogue between gut and bone offers new targets for therapeutic intervention that could potentially address both intestinal inflammation and its skeletal consequences simultaneously.

As our understanding of this complex signaling network continues to evolve, the GDS pathway stands as a paradigmatic example of how molecular insights from experimental models can illuminate the systemic nature of IBD and identify novel therapeutic targets for its extraintestinal manifestations [44]. The complexity of GDS signaling across multiple organ systems presents significant challenges for developing targeted interventions for skeletal complications in IBD, requiring thorough validation through multiple model systems, dose-response studies, and careful assessment of off-target effects. While GDS represents one critical mediator in the gut-bone axis, vitamin D signaling emerges as another key pathway that simultaneously influences intestinal inflammation, bone metabolism, and muscle function in IBD.

### Vitamin D signaling in IBD-related bone and muscle pathology

#### Immunomodulatory effects of vitamin D in IBD

The vitamin D and vitamin D receptor (VDR) complex plays a crucial role in modulating both innate and adaptive immune responses. Vitamin D helps reduce inflammation in experimental models of IBD and IBD patients, thereby contributing significantly to intestinal homeostasis [104]. Vitamin D exerts immunosuppressive effects through multiple mechanisms, including inhibition of toll-like receptors, TNF-α and IL-12 expression, reduction of T cell activation, enhancement of IL-10 production, and promotion of regulatory T cells [105]. Additionally, vitamin D impairs B cell development and suppresses monocyte activity. Also, vitamin D stimulates the production of various antimicrobial peptides, including β-defensins, cathelicidin, butyrate, and lithocholic acid, which collectively promote epithelial healing and contribute to anti-inflammatory effects [106, 107]. Studies utilizing VDR/IL-10 double KO mice and other experimental models have provided crucial insights into vitamin D’s role in maintaining intestinal barrier function. These models demonstrate that double KO mice develop colitis by 8 weeks, while epithelial VDR induction reduces disease activity [108]. Vitamin D’s specific effects on barrier function encompass regulation of tight junction proteins, enhancement of antimicrobial defenses, and upregulation of claudin-2 in DSS colitis models. Furthermore, vitamin D effectively prevents bacterial infiltration and promotes the restoration of normal histology when administered as cholecalciferol. These protective mechanisms on intestinal barrier integrity connect to vitamin D’s broader role in gut homeostasis.

#### Bidirectional relationship between vitamin D and gut microbiota

Vitamin D’s role in IBD extends beyond direct immunomodulation to include significant interactions with the gut microbiome. Notably, vitamin D and gut microbiota engage in a bidirectional interaction [106]. In one direction, VDR activation can modulate the composition and function of gut microbiota, which subsequently affects calcium absorption and vitamin D metabolism. This represents a regulatory effect of vitamin D signaling on the microbial community. In the other direction, the gut microbiota can influence vitamin D downstream pathways, potentially by producing metabolites that interact with the VDR or by affecting enzymes involved in vitamin D metabolism [106]. This bidirectional interaction creates a feedback loop where alterations in one system (vitamin D signaling or gut microbiota) can significantly impact the other, with important implications for intestinal homeostasis and inflammatory conditions like IBD. This relationship helps explain why vitamin D deficiency is often observed in IBD patients and why vitamin D supplementation can have beneficial effects beyond simply addressing the deficiency. By maintaining gut microbial balance, vitamin D creates a protective environment against dysbiosis and subsequent inflammatory processes that characterize IBD. Inadequate vitamin D levels alter microbial communities, weaken barrier function, and enhance susceptibility to inflammatory damage—processes that may further impair vitamin D metabolism and absorption in the diseased intestine. The efficacy of this protection, however, can be compromised by vitamin D deficiency, which itself may be exacerbated by environmental factors including diet, age, and genetics [109].

Vitamin D supplementation has been found to enhance Bacteroides and Parabacteroides populations, which are often diminished in IBD patients. Additionally, research on VDR-deficient mice reveals significant gut microbiota dysregulation, marked by reduced beneficial Lactobacillus and increased pathogenic Clostridium and Bacteroides. Notably, Parabacteroides has been identified as a significant microbial species closely linked to the human VDR gene. Treatment with 1,25-dihydroxyvitamin D3 demonstrates therapeutic potential by alleviating colitis severity and reducing Helicobacteraceae abundance in the fecal microbiome of cytochrome P450 (Cyp) KO mice [109]. Mechanistically, VDR and Cyp KO mice demonstrated compromised gut homeostasis characterized by reduced E-cadherin expression in epithelial and immune cells and impaired immune cell populations, significantly lower tolerogenic dendritic cell numbers, and compromised immune regulatory mechanisms. Consequences of these molecular changes included increased gut inflammatory responses, enhanced pathogen proliferation, disruption of microbial ecosystem balance, and reduced representation of beneficial microbiota. These findings conclusively demonstrate that deficiencies in 1,25(OH)2D3 or VDR directly contribute to gut microbiome dysbiosis, heightened susceptibility to intestinal injury, and compromised mucosal barrier integrity. Mice lacking Cyp27b1 (Cyp27b1^−/−^) exhibited chronic 1,25(OH)2D3 deficiency, whereas a 1,25(OH)2D3-deficient diet-induced acute deficiency, potentially leading to distinct effects on gut microbiota composition [110]. The research highlights the critical role of vitamin D signaling in maintaining gut homeostasis and immune system equilibrium.

#### Vitamin D effects on bone metabolism

Experimental evidence from colitis models suggests that vitamin D supplementation not only supports bone health directly but may also ameliorate intestinal inflammation and restore gut barrier function, indirectly benefiting the skeletal system [111]. Vitamin D stimulates RANKL expression while inhibiting OPG release from pre-osteoblasts, contributing to bone remodeling, and affects osteoclast precursor adhesion through ICAM-1. Pro-inflammatory cytokines released from inflamed intestinal mucosa, including IL-1, IL-6, and TNF-α, stimulate RANKL expression and enhance osteoclastogenesis [112]. Normal functioning of regulatory T cells depends on adequate vitamin D and VDR. Experimental evidence indicates that reduced vitamin D levels and impaired VDR signaling contribute to IBD development by increasing the production of pro-inflammatory cytokines such as TNF-α and interferon-γ [113]. However, high-dose vitamin D supplementation in DSS-induced colitis models has been associated with a shift toward a more inflammatory fecal microbiome profile [114]. Additionally, high-dose vitamin D3 supplementation resulted in deterioration of trabecular bone without impacting cortical bone morphology in the IL-10^−/−^ CD4^+^ T cell transfer model of chronic colitis [115]. Bone formation markers (osteocalcin and bone alkaline phosphatase) were decreased, and bone resorption markers, the ratio of RANKL to OPG transcript, plasma OPG level, and osteoclast activation marker (tartrate-resistant acid phosphatase) were increased. These adverse effects on bone health with high vitamin D3 doses likely reflectsynergistic interactions with circulating pro-inflammatory mediators that augment bone resorption [115].

#### Vitamin D effects on muscle metabolism

The discovery of VDR expression in skeletal muscle tissue has highlighted vitamin D’s direct effects on muscle metabolism and function, beyond its classical roles in calcium homeostasis [116, 117]. Skeletal muscle expresses both VDR and the vitamin D activating enzyme 1α-hydroxylase, enabling local vitamin D signaling [118]. Inflammatory cytokines can alter VDR expression in muscle tissue, potentially contributing to muscle weakness and atrophy [119]. Studies in VDR knockout mice have demonstrated reduced muscle fibre size and impaired muscle development, indicating vitamin D’s essential role in maintaining muscle mass and function [116, 120].

Vitamin D regulates muscle protein homeostasis through multiple mechanisms that become particularly relevant in the context of IBD-associated muscle wasting. The hormone promotes protein synthesis via activation of the mTOR signaling pathway while simultaneously reducing protein degradation by suppressing ubiquitin-proteasome activity [121, 122]. Additionally, vitamin D regulates myogenic differentiation through modulation of myogenic regulatory factors and influences muscle stem cell (satellite cell) function and regenerative capacity [123]. In colitis models, vitamin D deficiency exacerbates muscle protein breakdown and impairs regenerative responses, contributing to the development of sarcopenia [124]. These findings establish vitamin D insufficiency as a critical contributor to muscle wasting in IBD, suggesting that vitamin D supplementation may represent a therapeutic strategy that simultaneously addresses intestinal inflammation, bone deterioration, and muscle atrophy—providing a comprehensive approach to managing IBD-associated osteosarcopenia.

### Clinical relevance and translational implications

The experimental colitis models and mechanistic pathways discussed in this review have significant translational implications for understanding and managing IBD-associated osteosarcopenia in humans. Here, we examine how key findings from animal studies may inform clinical approaches to prevention, diagnosis, and treatment.

#### From experimental colitis to human IBD pathophysiology

Findings from DSS, TNBS, and genetic models demonstrate inflammatory pathways that appear to be conserved in human IBD pathophysiology. Clinical studies have confirmed similar patterns of pathways in IBD patients with CD and UC who develop musculoskeletal complications. For example, At the cytokine level, the pro-inflammatory mediators driving bone and muscle deterioration in colitis models—particularly TNF-α, IL-1, IL-6, and IL-23—have been independently validated in human IBD patients with musculoskeletal complications. In human studies, these cytokines work synergistically to enhance receptor activator of NF-κB ligand (RANKL)-induced signaling in osteoclasts through NF-κB and MAPK pathways, mirroring findings from experimental systems. TNF-α’s inhibition of R-spondin 2 (RSPO2), a protein crucial for osteoblast development, has been documented in both rodent models and human tissue samples [125]. Similarly, IL-23 released by macrophages and dendritic cells promotes osteoclastogenesis through the RANK/RANKL pathway in both experimental models and human IBD patients [66].

In IBD patients, increased pro-inflammatory cytokines (TNF-α, IL-1, IL-6, and IFN-γ) and lower levels of IGF-1 disrupt the balance between anabolism and catabolism, shifting it towards muscle atrophy [126]. This inflammatory environment, coupled with reduced anabolic signalling, exacerbates muscle degradation in IBD patients, contributing to sarcopenia [127].

Muscle atrophy is driven by lower IGF-1 levels and increased pro-inflammatory cytokines that activate the ubiquitin-proteasome system, particularly the atrogene and MURF-1 ubiquitin ligases [128]—precisely the same pathways implicated in experimental colitis models. The suppression of anabolic pathways including mTORC1 and MyoD transcription observed in animal studies is similarly documented in muscle biopsies from IBD patients. IL-6’s multifaceted contribution to muscle wasting—through reduced IGF-1, insulin resistance, decreased muscle mass, and increased fatigue—represents another conserved pathway between experimental models and human disease [129, 130].

However, important limitations must be acknowledged when extrapolating from experimental models to human disease. Human IBD represents a heterogeneous group of disorders with complex genetic, environmental, and immune components that cannot be fully recapitulated in animal models. The chronicity of human disease, often spanning decades, differs substantially from the relatively acute experimental models typically studied. Additionally, comorbidities, medication effects, dietary factors, and lifestyle variables present in human patients add layers of complexity absent in controlled experimental settings.

#### Serotonin signaling as a therapeutic target

The dysregulated serotonergic signaling observed in experimental colitis models closely parallels alterations documented in human IBD patients. Clinical evidence shows that elevated serum GDS levels have been conclusively demonstrated in UC patients [131], while altered expression of serotonin transporters and receptors characterizes both CD and UC. Specifically, patients with CD show upregulated expression of 5-HTR3, 5-HTR4, and 5-HTR7 in colonic tissues [132, 133]. In contrast, UC patients exhibit reduced serotonin transporter mRNA and protein levels in the colon tissues [132]—findings that mirror animal models where serotonin transporter deficiency directly causes significant bone loss [134]. These aberrations in tryptophan metabolism further contribute to intestinal inflammation through altered expression of antimicrobial peptides, compromising mucosal defense mechanisms and bacterial homeostasis [135].

The abundant presence of GDS in villi and lamina propria of IBD patients suggests its pathogenic role extends beyond intestinal symptoms to systemic effects [136, 137]. Although direct evidence specifically linking GDS to muscle wasting in human IBD remains limited, relevant pathways have been identified in related conditions that provide valuable insights into potential mechanisms [138, 139].

Clinical evidence regarding serotonin-bone relationships shows contradictory findings [91]. While Modder et al. demonstrated inverse associations between serum serotonin and total body/spine BMD in postmenopausal women [140], subsequent studies in healthy cohorts yielded inconsistent results [141]. Notably, patients with carcinoid syndrome, despite exhibiting markedly elevated serotonin levels, show no significant BMD alterations—suggesting possible receptor desensitization mechanisms [142]. Currently, no clinical studies have specifically examined this relationship in IBD patients, leaving the connection between circulating serotonin and bone density in this population unexplored despite compelling experimental evidence. In patients with IBD, many confounding factors such as diet, nutrient malabsorption, and corticosteroid exposure may affect bone metabolism in addition to inflammation, complicating the isolation of serotonin’s specific effects on skeletal outcomes in this population [143].

Research in related conditions has shown that peripheral serotonin metabolism influences kynurenine conversion during exercise—a process regulated by proliferator-activated receptor-gamma coactivator (PGC1α), which is frequently disrupted in inflammatory states [138, 139]. Additionally, increased expression of transcription factor FoxO1 in intestinal tissue of IBD patients [144] represents another potential mechanistic link, as FoxO1 modulates both inflammatory signaling and muscle atrophy pathways. The documented ability of FoxO1 to regulate TLR4/MyD88/MD2-NF-κB signaling and intestinal permeability in human IBD creates a plausible pathway connecting intestinal inflammation to systemic musculoskeletal effects.

While confirmation of HTR1B receptor expression on osteoblasts in human IBD patients remains pending, the conservation of serotonergic pathways across species suggests that the bone-protective effects of HTR1B antagonism observed in preclinical models may translate to clinical benefit. These converging lines of evidence indicate that targeting the GDS pathway could represent a novel therapeutic approach for addressing musculoskeletal complications in IBD patients, potentially preserving bone and muscle integrity even in the setting of ongoing intestinal inflammation.

Targeting the gut-bone axis through serotonin pathways represents a particularly promising frontier in managing IBD-associated bone loss. Peripheral serotonin inhibition, which proved effective in preventing bone loss in colitis models, may offer a novel therapeutic approach for osteoporosis, providing a unique intervention point in gut-bone signaling that operates distinctly from traditional anti-inflammatory approaches. While these preclinical findings show considerable promise, clinical validation in human studies remains a critical next step to confirm their translational potential.

#### Vitamin D interventions: from bench to bedside

Vitamin D emerges as a pivotal multifunctional mediator in IBD’s gut-bone-muscle axis, functioning simultaneously as an immune modulator, bone metabolism regulator, and myocyte function supporter. Clinically, high-dose 1,25(OH)₂D therapy demonstrates significant anti-inflammatory effects, reducing IL-17 A and IFN-γ levels while decreasing CD relapse risk [145–147]. Patients with elevated vitamin D levels before anti-TNF-α therapy show superior treatment longevity [148]. Beyond intestinal benefits, vitamin D directly regulates calcium absorption and the RANK/RANKL/OPG system critical for bone homeostasis [149].

The protective effects of vitamin D supplementation observed in colitis models align with epidemiological data showing improved BMD at lumbar spine and hip in CD patients [150], reduced fracture incidence in post-menopausal IBD patients [151], and showed potential for reversing skeletal damage in pediatric populations [152]. Standard clinical practice includes prophylactic vitamin D during glucocorticoid therapy to prevent bone deterioration [153, 154].

Combination therapy of maintained vitamin D levels with regular exercise effectively prevents muscle mass reduction in CD patients while improving hip muscle strength and reducing fall risk [126, 155, 156]. Despite the high prevalence of sarcopenia in CD patients and the established link between vitamin metabolism and muscle function, research examining the intersection of these three conditions remains limited [155].

While interest in this area is growing, particularly in paediatric populations [157], comprehensive studies are scarce. Currently, only one notable study by Hradsky et al. has investigated this relationship, demonstrating improved muscle parameters following vitamin D supplementation in IBD patients [158]. However, this study had limitations, including that it did not differentiate between CD and UC and relied solely on muscle strength measurements to assess sarcopenia. Given our understanding of vitamin D’s molecular effects on muscle metabolism, vitamin D supplementation could potentially improve sarcopenia in CD patients, though this hypothesis requires further investigation.

#### Age-specific therapeutic considerations

The age-dependent differences in experimental colitis severity and musculoskeletal outcomes highlight the need for age-stratified approaches in human IBD management. The complex interplay between IBD, sarcopenia, and frailty creates a particularly challenging clinical scenario in elderly patients [159], where the relationship appears bidirectional—intestinal inflammation drives muscle and bone deterioration, while resulting osteosarcopenia further complicates disease management [160]. In elderly IBD patients with dual vulnerability to skeletal complications, sarcopenia accelerates bone mineral deterioration, creating a dangerous cascade that increases fracture risk, necessitates hospitalization, and limits physical mobility—further compromising quality of life and independence [125]. The risk of osteoporosis-related fractures weighs heavily on elderly IBD patients due to the detrimental combination of aging, menopausal changes in women, and disease-associated inflammatory processes, creating a perfect storm of skeletal vulnerability [161].

Elderly IBD patients face compounded risks due to the convergence of disease-related inflammation, age-related physiological changes, and postmenopausal bone loss in women. Clinical data confirm that elderly IBD patients experience distinct therapeutic responses. Vitamin D intervention efficacy shows distinct age-dependent patterns in IBD patients. Meta-analyses reveal modest but significant BMD improvements and fracture risk reduction specifically in post-menopausal women with IBD [151]. In contrast, pediatric and young adult patients (ages 5–21) demonstrate more robust skeletal recovery potential following vitamin D supplementation, suggesting enhanced bone restoration capacity in developing skeletons [152]. This age-dependent response pattern underscores the importance of early intervention with calcium and vitamin D supplementation, particularly in vulnerable demographics—post-menopausal women whose estrogen deficiency compounds inflammatory bone loss, malnourished individuals with compromised mineral absorption, and patients on glucocorticoid therapy who face accelerated bone catabolism [154]. These differing therapeutic responses highlight the need for age-tailored dosing strategies in clinical management.

Current treatment guidelines insufficiently address these age-specific concerns. Based on findings from emerging aged animal models, therapeutic interventions for elderly IBD patients may benefit from combination approaches targeting both inflammation and direct musculoskeletal support, and earlier intervention with bone-protective agents. These strategies would address their unique inflammatory profile and reduced musculoskeletal regenerative capacity, potentially improving outcomes for this vulnerable and growing patient population.

### Therapeutic approaches for IBD-associated osteosarcopenia

Controlling the underlying inflammatory processes in IBD represents the cornerstone approach to preventing and treating associated musculoskeletal complications. Effective anti-inflammatory interventions can interrupt the pathophysiological cascade that leads to osteosarcopenia by suppressing the production of pro-inflammatory cytokines, preserving intestinal barrier function, and normalizing bone and muscle metabolism. The timing of intervention is crucial, as early treatment may prevent the development of musculoskeletal deterioration, while later intervention can slow progression and potentially reverse established damage [162].

#### Control IBD inflammation

IBD is characterized by persistent, non-specific intestinal inflammation, shaping the landscape of therapeutic development. Consequently, most clinical investigations explore compounds with direct anti-inflammatory capabilities, particularly those targeting established inflammatory mediators such as TNF-α. Anti-TNF-α interventions preserve intestinal barrier integrity during active inflammation, potentially interrupting a critical pathway linking intestinal pathology to systemic complications including bone and muscle deterioration [163].

Anti-TNF-α therapy changed the therapeutic armamentarium to prevent bone loss in moderate to severe IBD [164–166]. They have demonstrated efficacy in reducing pro-inflammatory cytokines that drive osteoclastogenesis through the RANKL/OPG pathway. Additionally, by dampening inflammation, these treatments can relieve the inhibitory effects on osteoblast differentiation and function, potentially restoring bone formation capacity. Similarly, reducing inflammation can benefit muscle metabolism by inhibiting protein breakdown pathways that are pathologically activated by pro-inflammatory cytokines [167].

Despite the theoretical promise of anti-TNF therapies in addressing IBD-related bone complications, their widespread clinical implementation remains constrained by a critical shortage of high-quality clinical trials specifically examining bone outcomes. This evidentiary gap highlights the need for dedicated research to fully establish their efficacy in skeletal protection [168]. Clinical experience suggests that anti-TNF therapy can significantly slow the progression of musculoskeletal deterioration within 3–6 months of treatment initiation, with some patients experiencing partial recovery of BMD and muscle mass over longer treatment periods [169].

Newer biologics targeting alternate inflammatory pathways, including JAK inhibitors, such as tofacitinib and upadacitinib, have demonstrated considerable efficacy in controlling intestinal inflammation [170, 171]. Initial evidence suggests JAK inhibitors may provide more rapid suppression of osteosarcopenia compared to other biologics due to their direct inhibition of multiple inflammatory signaling pathways simultaneously, though long-term data on musculoskeletal outcomes are still emerging [172]. However, their potential impact on skeletal health represents an evolving area of investigation [173]. The osteological implications of JAK inhibition in IBD patients remain incompletely characterized, underscoring the need for dedicated skeletal endpoints in future clinical evaluations of these promising compounds.

#### IL-12/23 inhibition

The IL-12/23 pathway exhibits complex, sometimes contradictory effects on bone metabolism that may have significant implications for IBD-associated bone loss. IL-12 appears to inhibit osteoclastogenesis through a unique mechanism involving TNF-α, triggering apoptosis in bone marrow osteoclast precursors [174, 175]. In contrast, IL-23 demonstrates predominantly pro-resorptive effects either by stimulating IL-17 production, upregulating RANK expression, or enhancing osteoclastogenesis in osteoblast co-cultures This suggests that selective IL-23 inhibition, rather than dual IL-12/23 blockade, might offer optimal skeletal benefits [176, 177].

Ustekinumab, which targets both IL-12 and IL-23, potentially improves bone health by suppressing RANKL expression, though clinical evidence confirming this mechanism remains pending. As newer selective IL-23p19 inhibitors like risankizumab enter clinical use for IBD [178], their differential impact on skeletal outcomes compared to broader IL-12/23 inhibition warrants dedicated investigation [125].

Beyond its effects on bone metabolism, the IL-12/23 pathway also plays a critical role in regulating muscle homeostasis during intestinal inflammation. Inhibition of the IL-12/23 p40 subunit protected against muscle wasting in DSS-induced colitis models [37]. This protection likely stems from inducing apoptosis of pathogenic T cells in gut mucosa, thereby improving intestinal disease and reducing systemic inflammation. IL-23 was found to activate Erk signaling and increase myh7 gene expression in the tibialis anterior muscle [37]. This finding is particularly relevant as several IL-12/23 pathway inhibitors, including ustekinumab, are already approved for IBD treatment and may provide additional muscle-protective benefits [179], offering a potentially comprehensive approach for addressing both intestinal and extraintestinal manifestations with a single therapeutic intervention. These findings highlight how targeting specific inflammatory pathways can yield benefits beyond intestinal inflammation to address extraintestinal manifestations simultaneously.

While the above approaches target inflammatory pathways, direct modulation of bone metabolism represents another promising strategy for IBD-associated skeletal complications.

#### RANKL inhibition: a dual-action approach for IBD-associated complications

Denosumab, a RANKL inhibitor established for osteoporosis treatment, remains understudied in IBD populations despite potential relevance. Genomic studies identified a variant near the TNFSF11 gene, encoding RANKL, associated with risk of CD, suggesting RANKL’s involvement in IBD pathogenesis beyond its established role in bone metabolism [180].

Preliminary evidence shows promising results in animal models. Recombinant human osteoprotegerin (rhOPG), a soluble decoy receptor that binds and neutralizes RANKL, demonstrated bone-protective effects in a T cell-transfer model of colitis [40]. Prophylactic treatment with Fc-OPG increased BMD, inhibited osteoclast function, and restored serum calcium and phosphate concentrations in mice receiving either CD4^+^CD45RB^high^ or CD4^+^CD45RB^low^ T cells without affecting gut inflammation [40]. The treatment also partially corrected the hypoparathyroidism observed in CD4^+^CD45RB^high^ T cell recipients, though it did not affect the bone marrow inflammatory cell infiltrate expressing TNF-α.

Similar therapeutic effects were observed in IL-2^−/−^ colitis mice, where exogenous Fc-OPG administration reduced both bone loss and colitis by modulating RANKL-RANK interactions [43]. However, these findings await confirmation in human IBD cohorts. The drug appears to unexpectedly influence bone formation markers alongside its intended anti-resorptive effects. A significant consideration for the typically younger IBD population is the documented rebound effect following denosumab discontinuation, potentially limiting its utility as a long-term therapy for IBD-associated bone loss without careful management strategies [181].

#### Sclerostin inhibition: a promising target for IBD-associated bone loss

While RANKL inhibition primarily suppresses bone resorption, targeting bone formation pathways offers a complementary therapeutic approach for IBD-associated bone loss.

Sclerostin, a negative regulator of bone formation produced by osteocytes, has emerged as a potent target for osteoporosis treatment. Ablation of the sclerostin gene in mice leads to increased bone mass, confirming its central role in bone metabolism [182]. In experimental colitis models, prophylactic administration of sclerostin antibody (Scl-AbI) completely blocked the loss of femoral and total BMD without affecting intestinal inflammation [183].

When researchers administered Scl-AbI therapeutically in mice with established colitis-induced bone loss, the treatment not only prevented further deterioration but largely restored normal trabecular bone volume and thickness to levels similar to healthy controls. Importantly, these skeletal benefits occurred without altering gut inflammation, as assessed by clinical score, weight loss, histology, and cytokine levels [183].

Clinical studies with sclerostin antibodies demonstrate this same powerful mechanism, showing an initial robust increase in bone formation markers accompanied by a pronounced decrease in bone resorption markers—creating a dual anabolic and anti-resorptive effect. This combination drives substantial increases in BMD and translates to fracture reduction in human trials [184]. This compelling efficacy profile suggests sclerostin inhibition could effectively address the severe bone loss associated with IBD, even in cases where intestinal inflammation persists despite optimal gastroenterological management. The positive effects of sclerostin antibody treatment in experimental colitis-associated bone loss support the potential investigation of romosozumab in IBD patients with osteoporosis. Similarly, denosumab’s mechanism parallels the beneficial effects of recombinant OPG observed in colitis models.

#### MDL-1 antibody: target for refractory skeletal complications

Beyond targeting established bone regulatory pathways, novel inflammatory mediators specific to IBD-associated bone loss may offer additional therapeutic opportunities.

For IBD patients experiencing persistent bone loss or non-healing fractures despite optimal control of intestinal inflammation, targeting specific osteoclast pathways may provide additional skeletal protection. The myeloid DNAX activation protein 12-associating lectin-1 (MDL-1) axis represents a particularly promising target, as anti-MDL-1 antibody treatment effectively prevents colitis-induced bone loss by reducing abnormal osteoclast differentiation both in vitro and in vivo [57].

This targeted approach demonstrates the potential for developing therapies that specifically address the skeletal manifestations of IBD without necessarily altering intestinal inflammation, which might be especially valuable when combined with standard IBD therapies such as anti-cytokine monoclonal antibodies. Such combinations could comprehensively manage all aspects of IBD, including refractory musculoskeletal complications that can significantly impact patient quality of life even when gut symptoms are controlled. Future clinical investigations should evaluate these combination approaches to determine if MDL-1 blockade remains effective in reducing IBD-associated bone loss when integrated with standard care therapy.

#### Myokine irisin: exercise-mimetic with dual benefits

The myokine irisin demonstrated dual benefits in TNBS and DSS models of colitis. Irisin treatment alleviated both gut and bone inflammation, decreased TNF-α, increased bone formation, and decreased osteoclast surfaces, while simultaneously improving gut histopathology and bone turnover [33]. These findings highlight a novel approach potentially addressing both intestinal inflammation and skeletal deterioration, and emphasize the benefits of physical activity in IBD management.

While serum measurements of inflammatory mediators like RANKL and TNF-α provide valuable clinical insights, study allowed for direct assessment of tissue-specific inflammatory changes, circumventing the inherent variability of circulating markers [185]. The near-perfect conservation of irisin across mammalian species (nearly 100% sequence homology) suggests strong translational potential for human applications. Though not yet in clinical trials for IBD, irisin has attracted interest as a potential treatment for metabolic disorders due to its ability to convert white adipose tissue to brown adipose tissue, increasing energy expenditure. This established metabolic profile may accelerate its development for treating IBD and its musculoskeletal complications [185].

#### Growth hormone signaling: tissue-specific regulation

Growth hormone (GH) signaling exhibits complex, tissue-specific effects in IBD, with suppressor of cytokine signaling 2 (SOCS2) emerging as a critical negative regulator of this pathway. In a DSS-induced colitis model, SOCS2 knockout (SOCS2^−/−^) mice demonstrated a paradoxical phenotype: increased intestinal disease activity with elevated pro-inflammatory cytokines (NOS2, IL-1β) during active inflammation, yet enhanced recovery with reduced fibrosis, decreased TGF-β1 receptor expression, and increased epithelial proliferation during the healing phase SOCS2 appears to restrain initial inflammatory responses while potentially limiting epithelial regeneration during recovery [186].

Most remarkably, SOCS2 deficiency conferred significant protection against colitis-induced bone loss. While DSS treatment in wild-type mice caused substantial deterioration of trabecular architecture (decreased bone volume, trabecular thickness, trabecular number, and increased separation), SOCS2−/− mice showed partial skeletal preservation with minimal changes in trabecular thickness and separation. This bone-protective effect appeared independent of intestinal disease severity, suggesting direct enhancement of GH action on bone tissue rather than secondary improvements in gut inflammation [187].

These findings reveal SOCS2 as a pleiotropic regulator with distinct tissue-specific roles during inflammatory states. In the intestine, SOCS2 appears to restrain initial inflammatory responses while potentially limiting epithelial regeneration during recovery. In contrast, its absence in bone tissue allows unregulated GH/IGF-1 signaling that maintains skeletal integrity despite ongoing inflammation. This differential impact provides crucial mechanistic insights for developing targeted therapies that could enhance GH signaling selectively in bone while preserving its appropriate regulation in other tissues, potentially addressing the skeletal complications of IBD without exacerbating intestinal inflammation [167, 188].

#### Extracellular vesicles-based approaches

Moving beyond traditional pharmaceutical interventions, cell-free therapeutic approaches offer new possibilities for targeted treatment of IBD-associated osteosarcopenia.

Extracellular vesicles (EVs)-based therapies targeting the disrupted bone marrow niche in IBD are currently advancing through preclinical and early clinical studies [189–191]. These natural nanocarriers demonstrate several advantages over conventional therapeutics: ability to cross biological barriers intact, target specific tissues, and deliver multiple bioactive molecules [192]. Modified EVs carrying anti-inflammatory drugs show enhanced tissue targeting and therapeutic efficacy in preclinical models, suggesting potential applications for treating refractory disease [192].

Guo et al. demonstrated that bone-targeted drug delivery to bone marrow stromal cells via exosomes can restore their proliferation and osteogenic capacity by redirecting differentiation toward osteoblasts. Exosomes with Golgi glycoprotein 1 inserted could carry Wnt agonist 1 and accumulate in bone via intravenous administration. They could alleviate bone loss, promote bone formation, and accelerate fracture healing in colitis mice [75]. The exosome-based drug delivery systems demonstrated in colitis mice leverage technologies already in clinical development for other conditions, suggesting a potentially rapid translation pathway.

Extracellular vesicles (EVs) represent a clinically promising approach for IBD management [192]. Initial clinical investigations show that EVs can modulate key inflammatory pathways without systemic side effects. Current evidence remains limited to preclinical studies, where EV-mediated delivery of bone protective factors has demonstrated encouraging results in experimental colitis models.

#### Prebiotics and microbiome-based interventions

While direct targeting of inflammatory and bone metabolic pathways offers therapeutic potential, addressing the microbial dysbiosis underlying IBD represents another promising strategy for preventing osteosarcopenia.

The therapeutic potential of gut microbiome modulation represents another frontier in suppressing inflammatory drivers of osteosarcopenia. Microbial metabolites, particularly SCFAs produced through bacterial fermentation of dietary fibers, likely function as key messengers in IBD [193, 194]. Emerging research suggests that microbiota-targeted interventions may offer promising strategies for simultaneously addressing both intestinal inflammation and its musculoskeletal manifestations.

Probiotics represent one of the most extensively studied microbiota-based therapeutic approaches with potential benefits for the gut-bone-muscle axis [195]. Recent advances in IBD treatment have highlighted the therapeutic potential of intestinal probiotics, with specific strains including *Lactobacilli*,* Bifidobacteria*, and *Saccharomyces boulardii* showing particular promise. These beneficial microorganisms appear to mitigate IBD-induced osteoporosis and attenuate bone loss through modulation of key inflammatory regulatory mechanisms, particularly by enhancing T-regulatory cell activity and promoting the production of SCFAs [195, 196].

Various probiotic strains can suppress inflammatory states by modulating pro- and anti-inflammatory interleukins [197], with direct evidence of protective effects on both IBD-induced gut inflammation and bone health [198]. Administration of *Lactobacillus reuteri* has been shown to prevent bone loss in animal models through the WNT10B pathway [199], while simultaneously decreasing intestinal inflammation and increasing trabecular bone volume fraction, bone mineral density, and bone mineral content in both vertebral and femoral sites through its anti-TNFα properties [200].

In experimental models, DSS-induced colitis caused increased gut permeability in female BALB/c mice, which was prevented with *Bifidobacterium longum* CCM 7952 treatment [201]. However, probiotics appear to have differential effects on bone inflammation, as treatment of bone marrow-derived dendritic cells from mice with VSL#3 showed increases in both pro- and anti-inflammatory cytokine levels [202].

Beyond bone health, the reduction of high gut permeability through probiotic supplementation may contribute to ameliorating muscle wasting [203]. Although studies specifically addressing muscle complications in the context of IBD are limited, reducing gut permeability via probiotic supplementation represents a potential treatment strategy for preserving muscle mass and function. Prebiotics complement the effects of probiotics by enhancing the production of SCFAs that support intestinal barrier integrity and modulate immune responses critical for both bone and muscle health [204].

Emerging research suggests specific SCFA-producing bacteria like *Clostridium butyricum* (CBM 588) could be valuable in gut-bone axis investigations, given butyrate’s established benefits for bone formation. Secondary bile acids, which are produced through bacterial modification of host-derived primary bile acids, represent an emerging therapeutic target with multisystem benefits [205].

Recent clinical studies have revealed that IBD patients exhibit intestinal dysbiosis accompanied by significantly reduced excretion of secondary bile acids, establishing a clear association between bile acid metabolism and disease pathogenesis [17, 204]. The therapeutic potential of secondary bile acids stems from their remarkable ability to mimic exercise-induced metabolic benefits. Deoxycholic acid (DCA) and lithocholic acid (LCA), the two principal secondary bile acids, activate G protein-coupled bile acid receptor 1 (GPBAR1) and farnesoid X receptor (FXR) signaling pathways, promoting mitochondrial biogenesis, uncoupling protein 1 (UCP1) expression, enhanced glucose metabolism, and reduced inflammation [205].

This finding is particularly relevant for addressing sarcopenia in IBD patients. Probiotic interventions that showed benefits in experimental colitis models have begun to show promise in human studies. Although clinical evidence specifically addressing skeletal outcomes remains limited, probiotics have demonstrated immunomodulatory effects in human IBD patients similar to those associated with bone and muscle protection in animal models.

Collectively, these diverse therapeutic approaches offer multiple strategies for preventing and treating IBD-associated osteosarcopenia. From established bone-targeted therapies like denosumab and sclerostin antibodies to novel approaches including myokine irisin, extracellular vesicles, and microbiome modulation, the therapeutic landscape continues to expand.

#### Natural compounds

Natural products and herbal medicines have demonstrated increasing promise for addressing both intestinal inflammation and associated skeletal complications [206]. These compounds often offer multi-target mechanisms that can simultaneously affect intestinal pathology and bone metabolism, potentially providing complementary approaches to conventional pharmaceuticals. Several compounds have shown particular promise in experimental models.

Oxyberberine’s displays remarkable ability to preserve bone integrity during active colitis. Daily administration not only reduces colonic damage but significantly maintains bone mass, strength, and microarchitecture—parameters typically compromised in untreated IBD. Mechanistically, oxyberberine disrupts the inflammatory cascade that triggers pathological bone resorption, effectively blocking the RANKL/NF-κB signaling pathway essential for osteoclast formation. This dual-action therapeutic—addressing both intestinal inflammation and skeletal deterioration—opens promising avenues for comprehensive IBD management where bone protection remains an underaddressed clinical need [168].

Dihydroartemisinin (DHA), a derivative of the traditional Chinese herb *Artemisia annua*, exhibits significant protective effects against experimental colitis and associated bone loss [207]. Artemisinin derivatives show promise in experimental colitis by simultaneously inhibiting NF-κB signaling, preserving intestinal barrier integrity, rebalancing T cells, and modulating gut microbiota. DHA effectively shifts immune responses from pro-inflammatory to regulatory phenotypes while promoting beneficial M2 macrophage polarization [208].

In DSS-induced IBD rat models, DHA effectively suppresses excessive osteoclast formation in bone tissue while simultaneously attenuating intestinal inflammation. Mechanistic studies suggest this dual benefit stems from DHA’s inhibitory effects on TNF-α signaling and RANKL expression, both critical mediators in the gut-bone inflammatory axis [207]. These findings highlight DHA is a potential therapeutic agent that could simultaneously target intestinal pathology and prevent skeletal deterioration in IBD patients, offering a complementary approach to conventional pharmaceuticals.

Beyond single compounds, traditional herbal formulations have also shown efficacy in experimental IBD models. Jianpi Qingchang Bushen Decoction (JQBD), a traditional Chinese herbal formulation, [209]. JQBD formulations have been used in human patients for centuries, albeit requiring rigorous modern clinical evaluation.

New insights regarding plant-derived flavonoid product naringin show alleviation of DSS-induced colitis symptoms, including disease activity index, colon length shortening, and colon pathological damage [210]. Naringin enhances the process of proliferation and differentiation of osteoprogenitor cells into osteoblasts while simultaneously inhibiting osteoclastic activity [211].

Naringin enhances the recovery of bone by promoting osteoblastogenic potential and inhibiting osteoclastogenesis to achieve rapid reconstitution of bone health in models of colitis. This compound prevents bone loss and diminishes the disease activity in the glucocorticoid-treated rat model of colitis [212]. In Sprague-Dawley rats, administration of naringin together with dexamethasone, startingin the second week after DSS-induced colitis, leads to a significant increase in the bone formation marker procollagen type I N-terminal propeptide. Naringin treatment up-regulates the expression of bone formation-related genes and reduces oxidative stress in bone from rats with dexamethasone-induced colitis. These findings suggest that naringin has substantial potential to promote bone formation [212].

Emodin, a type of anthraquinone compound, derived from the roots and bark of plants of the genus *Rhamnus*, has potential anti-inflammatory activities [56]. In one study, emodin was administered orally starting from the third week for 9 weeks in Sprague Dawley male rats with DSS-induced colitis [56]. The results showed significant deterioration of bone mass, biomechanical properties, microstructure parameters and an increased number of osteoclasts in the bone of rats with DSS-induced colitis. However, emodin intervention abolished these adverse changes in bone microstructure and biomechanical properties. Emodin also inhibited osteoclast formation and reduced serum levels of C-terminal cross-linked peptide (CTX) and TNF-α. Mechanistically, emodin significantly abolished the expression of TRAF6, NFATC1, and C-FOS associated with colitis. These findings demonstrate that emodin suppresses colitis-induced osteoporosis by inhibiting osteoclast formation, suggesting its potential as a natural therapeutic for IBD-associated bone loss [56].

Collectively, these natural compounds demonstrate promising effects on both intestinal inflammation and associated bone deterioration in experimental IBD models. Their multi-target actions and generally favorable safety profiles make them attractive candidates for translational investigation. While most evidence remains preclinical, the traditional use of many of these compounds in various medical systems suggests potential for clinical application. Future human studies will be crucial to determine optimal dosing, efficacy, and safety profiles of these natural compounds as complementary approaches for managing IBD-associated osteosarcopenia.

## Conclusions

This comprehensive review of experimental IBD models has revealed the complex and multifaceted nature of osteosarcopenia in chronic intestinal inflammation. Through examining a spectrum of models—from chemical induction to genetic manipulation and spontaneous disease—we have identified several key principles that enhance our understanding of this extraintestinal manifestation. 

First, the temporal relationship between intestinal inflammation and skeletal deterioration follows a consistent pattern across most models. Notably, multiple models demonstrate similar patterns of bone loss despite different mechanisms of colitis induction, suggesting common inflammatory pathways linking intestinal inflammation to skeletal deterioration [213]. The observation of specific inflammatory mediators in bone tissue—including G-CSF, TNF-α, IL-12p40, and various chemokines—provides mechanistic insights into how distant intestinal inflammation can trigger osteoclastogenesis and bone loss [213], creating opportunities for targeted therapeutic intervention.

Second, our analysis has revealed that multiple pathogenic mechanisms operate simultaneously to drive skeletal deterioration. Pro-inflammatory cytokines (particularly TNF-α, IL-6, and IL-17), gut-derived mediators (especially serotonin), altered vitamin D signaling, and disrupted calcium homeostasis create a complex network of pathways leading from intestinal inflammation to distant skeletal effects. This complexity explains why targeting individual pathways may yield only partial benefits and suggests that comprehensive therapeutic approaches will be necessary for optimal patient outcomes.

Third, the reciprocal relationship between bone and muscle deterioration, evident across multiple models, supports the clinical concept of osteosarcopenia as an integrated pathophysiological process rather than separate entities. The shared inflammatory mediators affecting both tissues explain the frequent co-occurrence of bone and muscle deficits in IBD patients and highlight the need for holistic approaches to skeletal health that address both components simultaneously.

Fourth, several models have revealed gender differences in susceptibility to both intestinal inflammation and skeletal deterioration, particularly the stronger muscle wasting phenotype observed in male mice with *H. hepaticus*-infected IL-10^−/−^ colitis. These findings parallel clinical observations of gender-specific musculoskeletal manifestations in IBD patients and emphasize the need for sex-specific approaches in both research and treatment strategies.

Fifth, age emerges as a critical modifier of inflammatory and skeletal pathways, potentially necessitating tailored therapeutic approaches for different patient demographics. While animal models have provided valuable mechanistic insights, further clinical investigations—particularly in elderly populations—are essential to develop age-appropriate interventions that can effectively prevent and treat musculoskeletal complications in IBD patients across the lifespan.

Based on these key principles derived from experimental models, several promising research directions emerge for future investigation.

Systems biology approaches could map the complete signaling networks connecting intestinal inflammation to bone and muscle metabolism, revealing new therapeutic targets and biomarkers. The gut-derived serotonin pathway presents particularly significant potential for intervention, with studies needed to evaluate tissue-specific effects of TPH1 inhibition and selective 5-HTR1B antagonism in human subjects.

Translational research should focus on validating key findings from animal models in human patients. This includes developing reliable biomarkers that predict skeletal complications and addressing the critical needs of pediatric IBD patients whose skeletal development may be permanently affected by inflammatory processes. The development of combination therapies that simultaneously address intestinal inflammation and skeletal protection represents an important goal for improving patient outcomes.

Future clinical research must validate these promising experimental approaches in human subjects. Prospective studies examining the effects of approved IBD therapies on bone and muscle parameters are needed, as are clinical trials specifically designed to evaluate interventions targeting the musculoskeletal complications of IBD. Biomarker studies correlating molecular pathways identified in experimental models with clinical outcomes in diverse IBD populations will help bridge the translational gap and identify the most promising therapeutic targets for clinical development.

The integration of these translational insights into clinical practice will ultimately require a multidisciplinary approach involving gastroenterologists, rheumatologists, endocrinologists, and rehabilitation specialists. Together, these experts can develop comprehensive treatment strategies addressing both intestinal inflammation and its musculoskeletal manifestations. This collaborative approach is particularly important for managing complex cases such as elderly IBD patients with pre-existing age-related bone and muscle deterioration.

By continuing to refine experimental models and bridge the gap between laboratory findings and clinical application, we can develop integrated approaches that consider both intestinal and extraintestinal manifestations as interconnected aspects of a systemic inflammatory condition. This holistic perspective will lead to more effective interventions that not only control gut inflammation but also preserve musculoskeletal health and improve the quality of life for the millions of IBD patients worldwide who currently suffer from these debilitating extraintestinal complications.

## Data Availability

Not applicable.

## Abbreviations

BMD: Bone mineral density
CD: Crohn’s disease
DSS: Dextran Sodium Sulfate
EC: Enterochromaffin cells
EIM: Extraintestinal manifestations
GF: Germ-free
GDS: Gut-derived serotoninI
BD: Inflammatory bowel diseaseI
GF: Insulin growth factorIL Interleukin
LPS: Lipopolysaccharidem
TOR: Mammalian target of rapamycin
OPG: Osteoprotegrin
RANK: Receptor activator of nuclear factor-kappa BRANKL
RANK: ligandSCFAs Short-chain fatty acids
SCID: Severe combined immunodeficiency
SERT: Serotonin selective reuptake transporter
TLR4: Toll-like receptors 4
TNBS: 2, 4, 6 trinitrobenzene sulfonic acid
TNF: Tumor necrosis factor
TRP: Tryptophan
TPH1: Tryptophan hydroxylase 1
UC: Ulcerative colitis
VMAT: Vesicular monoamine transporter
